# *Pseudomonas syringae* pv. *syringae* Uses Proteasome Inhibitor Syringolin A to Colonize from Wound Infection Sites

**DOI:** 10.1371/journal.ppat.1003281

**Published:** 2013-03-28

**Authors:** Johana C. Misas-Villamil, Izabella Kolodziejek, Emerson Crabill, Farnusch Kaschani, Sherry Niessen, Takayuki Shindo, Markus Kaiser, James R. Alfano, Renier A. L. van der Hoorn

**Affiliations:** 1 Plant Chemetics Lab, Max Planck Institute for Plant Breeding Research, Cologne, Germany; 2 Center for Plant Science Innovation, University of Nebraska, Lincoln, Nebraska, United States of America; 3 School of Biological Sciences, University of Nebraska, Lincoln, Nebraska, United States of America; 4 Chemical Biology Group, Department of Biology, University Duisburg-Essen, Essen, Germany; 5 Department of Chemical Physiology, The Scripps Research Institute, La Jolla, California, United States of America; 6 Department of Plant Pathology, University of Nebraska, Lincoln, Nebraska, United States of America; The University of North Carolina at Chapel Hill, United States of America

## Abstract

Infection of plants by bacterial leaf pathogens at wound sites is common in nature. Plants defend wound sites to prevent pathogen invasion, but several pathogens can overcome spatial restriction and enter leaf tissues. The molecular mechanisms used by pathogens to suppress containment at wound infection sites are poorly understood. Here, we studied *Pseudomonas syringae* strains causing brown spot on bean and blossom blight on pear. These strains exist as epiphytes that can cause disease upon wounding caused by hail, sand storms and frost. We demonstrate that these strains overcome spatial restriction at wound sites by producing syringolin A (SylA), a small molecule proteasome inhibitor. Consequently, SylA-producing strains are able to escape from primary infection sites and colonize adjacent tissues along the vasculature. We found that SylA diffuses from the primary infection site and suppresses acquired resistance in adjacent tissues by blocking signaling by the stress hormone salicylic acid (SA). Thus, SylA diffusion creates a zone of SA-insensitive tissue that is prepared for subsequent colonization. In addition, SylA promotes bacterial motility and suppresses immune responses at the primary infection site. These local immune responses do not affect bacterial growth and were weak compared to effector-triggered immunity. Thus, SylA facilitates colonization from wounding sites by increasing bacterial motility and suppressing SA signaling in adjacent tissues.

## Author Summary

Bacterial plant pathogens are usually contained at wound infection sites by an effective immune response. The ways in which bacteria can overcome this spatial restriction is poorly understood. Here, we studied two *Pseudomonas syringae* pv. *syringae* strains that cause brown spot on bean and blossom blight on pear and are known to exist as epiphytes that cause disease upon wounding by hail, sand storms or frost. In this study, we demonstrated that these two strains suppress spatial confinement by producing a low molecular weight proteasome inhibitor, syringolin A (SylA), which acts in two ways. On one hand, SylA diffuses into tissue surrounding the primary infection site to make the tissue insensitive to immune signaling. On the other hand, SylA promotes bacterial motility and suppresses immune responses at the primary infection site. Consequently, SylA-producing bacteria are more motile and able to spread from the primary infection site through xylem vessels and colonize adjacent, immuno-compromised tissues along the vasculature.

## Introduction

Wounding of plants by hard wind, hail, heavy rain, sand storms, and frost is common in nature. Many epiphytic leaf pathogens take advantage of this opportunity to infect plants [1]. Although plants have evolved effective immune responses to protect wound sites, many pathogens are able to enter leaf tissues and cause disease [1]. The molecular mechanisms underlying the suppression of plant-mediated restriction of pathogen spreading from wound sites are poorly understood.

*Pseudomonas syringae* pv. *syringae* (*Psy*) causes brown spot on bean plants and blossom blight in pear trees [1], which are serious diseases responsible for significant yield losses in agricultural industries in the US, Africa, and Australia. *Psy* can grow epiphytically on leaf surfaces and enters the leaf intercellular space (apoplast) through stomata and wounds [1]–[3]. Upon entering the leaf apoplast, *Psy* initially propagates biotrophically, keeping the host cells alive, and later causes necrotic lesions [4]. Although *Psy* is a common leaf epiphyte, disease outbreaks are often seasonal and conditional [5]. For example, the onset of epidemics is associated with heavy rain storms and is related to raindrop momentum rather than an increase in humidity [6]. In addition, heavy wind without significant precipitation, causing damage by hail and blowing sand, has caused a brown spot outbreak causing 55% yield loss [7]. Likewise, although *Psy* strains are a common and dominant component of the microflora on pear trees, blossom blight disease only occurs after frost injury, which explains the strong seasonal variation of disease outbreaks [8]. These data illustrate that *P. syringae* takes advantage of natural wound sites to enter host tissue and cause disease. Thus, it is important to understand the molecular mechanisms underlying host entry at wound sites in order to prevent disease outbreaks. However, to date, these mechanisms have been poorly investigated.

We recently discovered that green fluorescent protein (GFP)-expressing *P. syringae* can escape from wound infection sites and colonize adjacent tissues in the wild tobacco plant *Nicotiana benthamiana* [9], which has become an important model plant for *P. syringae* infections [3], [10]–[16]. Colonies appeared up to 1 cm from the primary infection site within a few days. Although these infections are not systemic (throughout the whole plant), these distances, from the perspective of bacteria, are significant and increase the area of infection by several orders of magnitude. Using bacterial count assays with controlled inoculation populations, we have shown that bacterial populations can grow nearly 100-fold more if the bacteria colonize adjacent tissue compared to when they remain contained at the primary infection site [9]. The colonization from wound sites follows the vasculature, and electron microscopy experiments indicated that the bacteria move through xylem vessels [9]. The ability to colonize tissues along the vasculature involves four steps: first, the bacteria overcome local containment at the primary infection site; second, they transport themselves over several millimeters through the xylem; third, they escape from the xylem vessel into the apoplast; and finally, they colonize the apoplast in adjacent tissues. The molecular mechanisms underlying each of these events are not yet understood.

We found that colonization from wound sites on *N. benthamiana* is common for *P. syringae* strains representing the major branches of the *P. syringae* phylogenetic tree (phylogroups) [9]. Two of the strains that efficiently colonize tissues from wound sites are *P. syringae* pv. *syringae* B728a (PsyB728a) and B301D-R (PsyB301D), both from phylogroup II [16]. PsyB728a and PsyB301D cause brown spot on bean plants and blossom blight on pear trees, respectively, and both disease outbreaks occur upon wounding [6]–[8]. Besides approximately 30 type III effectors that manipulate the host cell [14], PsyB728a and PsyB301D also produce syringolin A (SylA), a small nonribosomal cyclic peptide that irreversibly inhibits the eukaryotic proteasome [17]–[18]. Compared to wild-type (WT) strains, SylA-deficient mutant strains of PsyB728a cause fewer brown spot symptoms on bean plants upon spray inoculation [18]. SylA-deficient mutants also display delayed entry into bean leaves, which led to the discovery that SylA suppresses preinvasive immunity by reopening stomata in bean plants and Arabidopsis [19]. Using proteasome activity profiling with proteasome-selective chemical probes, we have demonstrated that SylA preferentially inhibits the β2 and β5 catalytic subunits of the Arabidopsis proteasome and that SylA accumulates irreversibly in the nucleus, indicating that it targets the nuclear proteasome [20]. Thus, we hypothesized that the subunit and subcellular selectivity may be important parameters for the biological activity of SylA.

The aim of this study was to investigate the molecular mechanisms underlying wound entry by PsyB728a and PsyB301D. We demonstrate that both strains overcome spatial confinement at wound infection sites by producing SylA. SylA can diffuse from the primary infection site and block salicylic acid (SA) signaling in adjacent tissues, creating an SA-insensitive zone of vasculature tissue that is susceptible to secondary colonization. Furthermore, SylA was found to increase bacterial motility and suppress immune responses at the primary infection site. These local immune response do not affect bacterial growth and are weak compared to effector-trigged immunity (ETI) [21].

## Results

### The Δ*sylC* Mutant of PsyB728a Is Unable to Cause Spreading Lesions

**Figure 1:**
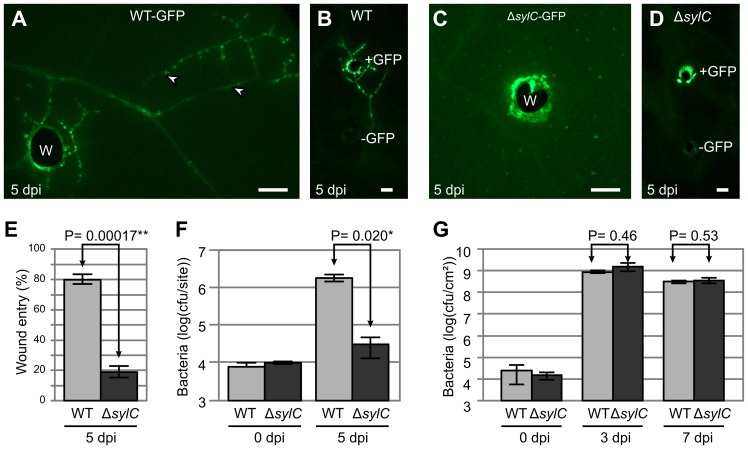
Wound entry by PsyB728a is hampered in the Δ*sylC* mutant. (**A–D**) Wound entry by WT but not *ΔsylC* PsyB728a. Untransformed and GFP-expressing WT and *ΔsylC* PsyB728a strains were wound-inoculated into *N. benthamiana*. Pictures were taken at 5 dpi by fluorescence microscopy using identical settings. W, wound site. Arrowheads indicate colonies in adjacent tissues. Scale bars, 1 mm. (**E**) WT bacteria spread more frequently from the wound inoculation sites than the Δ*sylC* mutant. The frequency of colonization of adjacent tissues was scored by fluorescence microscopy at 5 dpi. Error bars represent SEM of four independent experiments. (**F**) Bacterial population levels per wound inoculation site. Wound sites were immediately inoculated with 1 µL containing 10^4^ WT-GFP or Δ*sylC*-GFP bacteria. The bacterial titer was determined at 5 dpi in 8-mm diameter leaf discs containing the wound site with adjacent tissues. Error bars represent SEM of three extracts generated from three leaf discs each. This experiment was repeated twice with similar results. (**G**) Bacterial growth was indistinguishable between WT and Δ*sylC* bacteria upon infiltration. *N. benthamiana* plants were infiltrated with WT or Δ*sylC* mutant strains at 2×10^5^ bacteria/mL. Bacterial populations (in colony forming units (cfu) per cm^2^ leaf surface) were counted at different time points after infiltration. Error bars indicate SEM for four different samples. Similar results were obtained in repetition experiments with different inoculation densities, relative humidities, and sterilization procedures (see supplemental **Figure S1**). (**E–G**) P-values determined using the Student's *t*-test are indicated.

Pathovar *syringae* B728a is one of the strains that is able to enter wound sites and colonize adjacent tissues in *N. benthamiana* [9]. At 5 d after wound inoculation (5 dpi) with this strain, fluorescent colonies appeared along the vasculature at regular intervals (**Figure 1A**), which were absent in the untransformed control not expressing GFP (**Figure 1B**). To investigate the molecular mechanism underlying wound entry by PsyB728a of *N. benthamiana*, we tested various PsyB728a mutants for their ability to cause spreading lesions following toothpick inoculation. These assays revealed that wound entry by the GFP-expressing Δ*sylC* mutant of PsyB728a was strongly reduced, even though fluorescence was detected at the inoculation site (**Figure 1C**). This fluorescence at the inoculation site was caused by bacteria expressing GFP, since the untransformed Δ*sylC* control did not show this fluorescent signal (**Figure 1D**). Colonies of Δ*sylC* in adjacent tissues appeared in less than 20% of the wound inoculations, whereas WT PsyB728a showed colonization from wounding sites (wound entry) in over 80% of the inoculations (**Figure 1E**).

To demonstrate the relevance of spreading for bacterial growth, we inoculated wound sites with a controlled inoculum (1 µL containing 10^5^ bacteria) and determined the colony-forming units at 5 dpi in leaf discs containing the wound site and adjacent tissue. Under these conditions, WT bacterial populations grew from 10^4^ bacteria to over 10^6^ bacteria per infection site, whereas Δ*sylC* bacteria grew from the same inoculum population to about 3*10^4^ bacteria per inoculation site (**Figure 1F**). Thus, under these conditions, the ability to spread gives WT bacteria an advantage of 63-fold increased population growth compared to the Δ*sylC* mutant.

However, strong GFP fluorescence at the inoculation site (**Figure 1D**) suggested that Δ*sylC* bacteria were able to grow locally. Indeed, bacterial growth assays on infiltrated tissues did not show significant growth differences between WT and Δ*sylC* strains (**Figure 1G**). Furthermore, indistinguishable bacterial growth between WT and Δ*sylC* bacteria upon infiltration was also observed with different inoculation densities and at different relative humidity levels (Supplemental **Figure S1**). Although the Δ*sylC* strain sometimes grew less compared to the WT strain, the differences were only rarely statistically significant (3/16 comparisons with P<0.05 in **Figure S1**). The absence of a statistically significant growth phenotype upon infiltration is in contrast but not in conflict with earlier reports that the Δ*sylC* mutant causes less symptoms and less bacterial growth compared to WT bacteria upon spray inoculation [18]–[19], because SylA suppresses preinvasive immunity by reopening stomata [19]. Our data indicate that, once inside the leaf, WT and Δ*sylC* bacteria grow equally well at primary infection sites but Δ*sylC* bacteria are unable to colonize adjacent tissue.

### SylA Biosynthesis Is Necessary and Sufficient for Wound Entry by PsyB728a and PsyB301D

**Figure 2:**
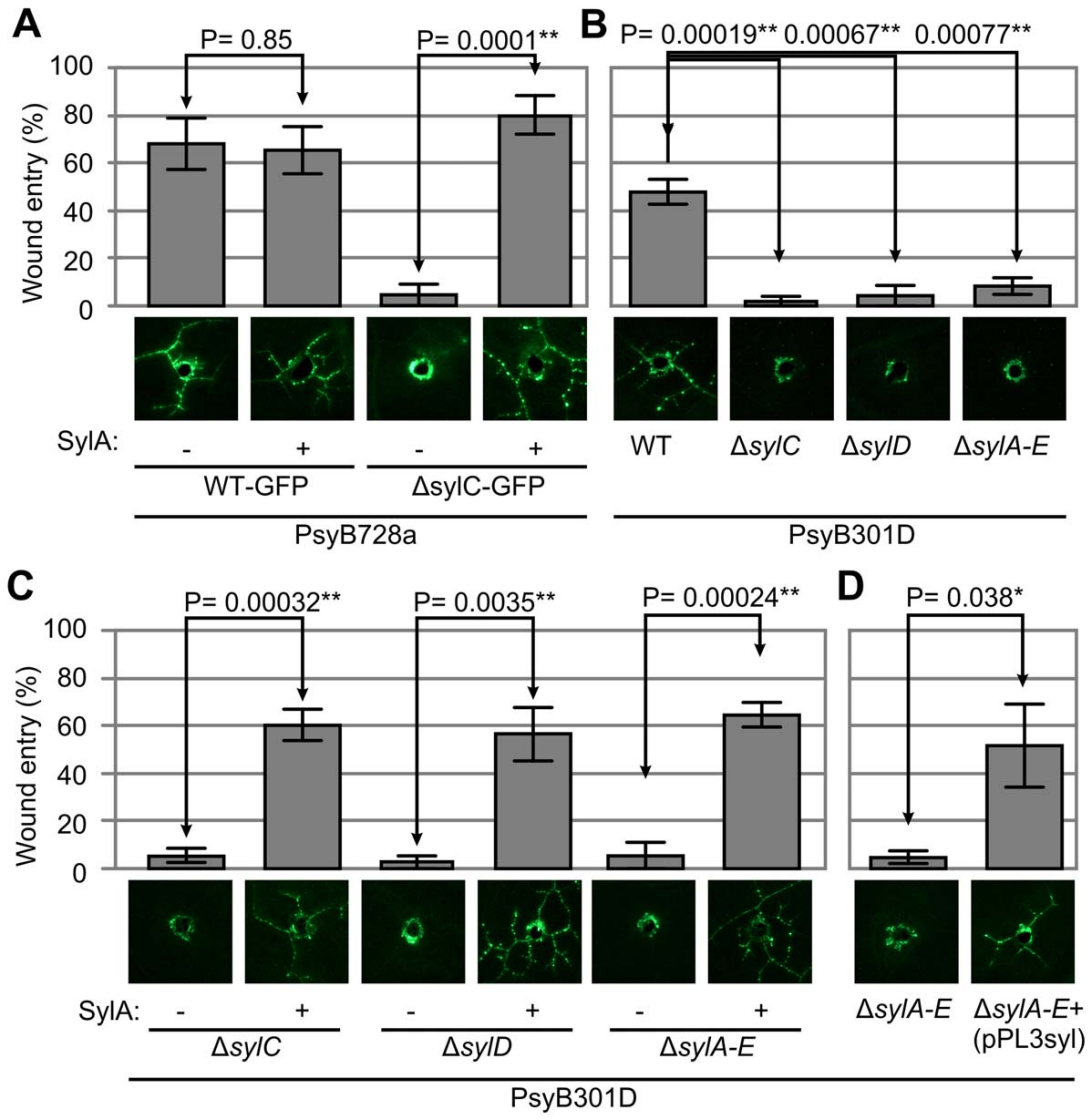
SylA is necessary and sufficient for wound entry of PsyB728a and PsyB301D. (**A**) Exogenous SylA complements wound entry of the SylA-deficient Δ*sylC* strain of PsyB728a. *N. benthamiana* leaves were infiltrated with 50 µM SylA or 0.25% DMSO and wound-inoculated 1 h later. Colonization was scored at 5 dpi by fluorescence microscopy. (**B**) SylA biosynthesis is necessary for wound entry by PsyB301D. GFP-expressing PsyB301D and derived mutants in the SylA biosynthesis clusters (Δ*sylC*, Δ*sylD*, and Δ*sylA-E*) were wound-inoculated and scored at 5 dpi by fluorescence microscopy. (**C**) Exogenous SylA complements wound entry of SylA-deficient strains of PsyB301D. *N. benthamiana* leaves were preinfiltrated with 50 µM SylA or 0.25% DMSO and wound-inoculated 1 h later. Colonization was scored at 5 dpi by fluorescence microscopy. (**D**) The SylA biosynthesis cluster complements wound entry by PsyB301D. The Δ*sylA-E* mutant of PsyB301D was transformed with cosmid pPL3syl carrying the SylA biosynthesis cluster. GFP-expressing derivatives were wound-inoculated, and colonization was scored at 5 dpi by fluorescence microscopy. (**A–D**) GFP-expressing strains were wound-inoculated into *N. benthamiana* leaves, and colonization of tissue adjacent to the wound site was scored at 5 dpi by fluorescence microscopy. The photographs at the bottom show representative pictures taken by fluorescence microscopy at 5 dpi. Error bars indicate SEM of four independent experiments, each with 12 inoculations. P-values determined using the Student's *t*-test are indicated.

To demonstrate that SylA itself is sufficient to promote wound entry, we wound-inoculated the GFP-expressing Δ*sylC* strain into tissue preinfiltrated with 50 µM SylA or a mock control. We used 50 µM SylA because PsyB301D is able to produce 40–100 µM SylA in cultures [17], and 50 µM SylA inhibits the host proteasome (see below). Exogenous SylA restored wound entry of the Δ*sylC* strain to a similar frequency as the WT strain (**Figure 2A**). In contrast, no wound entry by the Δ*sylC* strain was observed by infiltrating buffer without SylA (**Figure 2A**). These data demonstrate that SylA is essential and sufficient to promote wound entry by the bean brown spot pathogen PsyB728a.

To determine whether SylA biosynthesis is necessary for wound entry by PsyB301D, we tested SylA-deficient mutants of PsyB301D that lack different SylA biosynthesis enzymes (Δ*sylC* and Δ*sylD* mutants, [22]) or the entire SylA biosynthesis cluster (*ΔsylA-E*, [23]). Importantly, none of these mutant strains was able to colonize adjacent tissue (**Figure 2B**). The loss of wound entry could be complemented with exogenous SylA (**Figure 2C**), demonstrating that SylA is also essential and sufficient to promote wound entry by the pear blossom blight pathogen PsyB301D.

To test whether loss of the wound entry phenotype could be complemented by restoring SylA biosynthesis, we transformed the markerless PsyB301D *ΔsylA-E* mutant with the pPL3syl cosmid [23], which carries the entire SylA biosynthesis cluster. Transformation restored the capability to colonize adjacent tissues (**Figure 2D**), confirming that SylA biosynthesis genes are required to facilitate wound entry. The Δ*sylC* and Δ*sylD* mutants of PsyB301D and PsyB728a could not be transformed with the pPL3syl cosmid as these strains already contain the antibiotic selection marker that was used for the cosmid selection. Taken together, these chemical and genetic complementation assays demonstrate that SylA is required and sufficient to facilitate wound entry by PsyB728a and PsyB301D.

### SylA Targets the Proteasome of *N. benthamiana*

**Figure 3:**
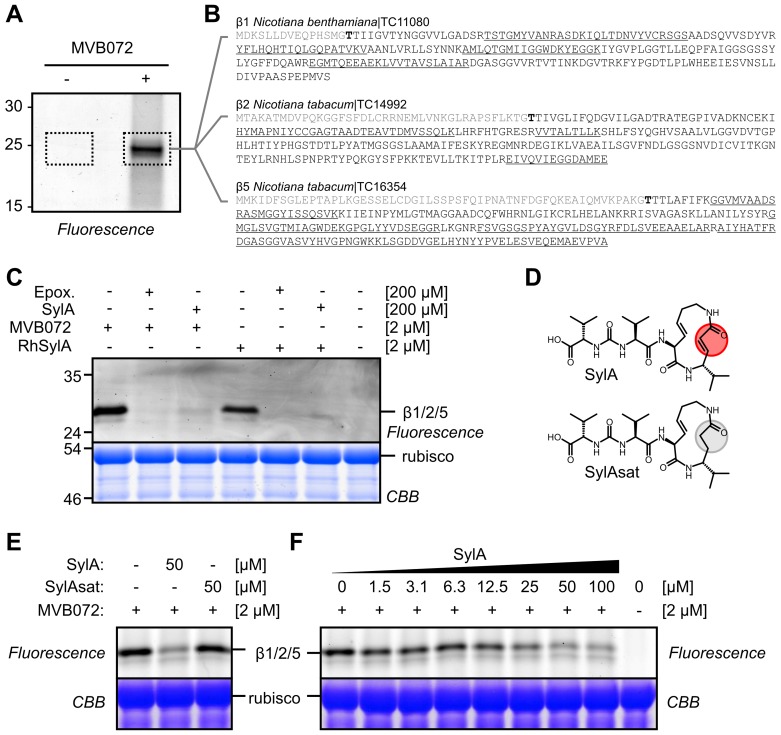
SylA targets the proteasome of *N. benthamiana*. (**A**) MVB072 labels the catalytic proteasome subunits in *N. benthamiana* leaf extracts. Leaf extract of *N. benthamiana* was incubated with or without MVB072, an epoxomicin-based probe carrying both biotin and BODIPY. Biotinylated proteins were purified and detected by in-gel fluorescence scanning. (**B**) Proteins identified by mass spectrometry. In-gel trypsin digests (dashed areas) were analyzed by tandem mass spectrometry. Identified peptides are underlined in the sequences of the β1, β2, and β5 catalytic subunits of the proteasome. None of these peptides were found in the no-probe-control. No peptides were from the propeptide (grey) or the mature N-terminus, containing the catalytic Thr (bold). (**C**) SylA targets the proteasome of *N. benthamiana*. Leaf extracts of *N. benthamiana* were preincubated with 200 µM epoxomicin (Epox.) or SylA for 30 min and then labeled for 2 h with 2 µM MVB072 or RhSylA. Labeled proteins were detected by in-gel fluorescence scanning, and proteins were stained with Coomassie blue. (**D**) Structures of SylA and SylAsat. SylA has a Michael system in the ring that reacts with the catalytic Thr residues of the proteasome. This Michael system is absent in SylAsat due to saturation of the double bond. (**E**) SylAsat does not fully inhibit the proteasome. Leaf extracts were preincubated with 50 µM SylA and SylAsat and labeled with MVB072. Proteins were separated on protein gels and detected by fluorescence scanning and Coomassie blue staining. (**F**) Concentration dependency of proteasome inhibition by SylA. Leaf extracts were incubated with various SylA concentrations and then labeled with MVB072. Proteins were analyzed on a protein gel using fluorescence scanning and Coomassie blue staining.

Next, we used proteasome activity profiling [20], [24] to investigate whether SylA inhibits the proteasome of *N. benthamiana*. Labeling of *N. benthamiana* leaf extracts with an activity-based probe for the proteasome (MVB072, Supplemental **Figure S2**) revealed a single 24 kDa band on protein gels (**Figure 3A**). Analysis of the purified labeled proteins by tandem mass spectrometry showed that this signal contained the β1, β2, and β5 proteins, the three catalytic subunits of the proteasome (**Figure 3B** and Supplemental **Figure S3**). Preincubation of *N. benthamiana* leaf extracts with SylA or the selective proteasome inhibitor epoxomicin blocked MVB072 labeling, indicating that SylA inhibited the proteasome of *N. benthamiana* (**Figure 3C**). Incubation of *N. benthamiana* leaf extracts with rhodamine-tagged SylA (RhSylA, [25], Supplemental **Figure S2**) revealed a single 24 kDa signal on protein gels, similar to MVB072 labeling (**Figure 3C**). Preincubation with SylA or epoxomicin blocked RhSylA labeling of these proteins, confirming that this signal represented the proteasome (**Figure 3C**). In contrast to SylA, no proteasome inhibition was detected upon preincubation with SylAsat (**Figures 3D** and **3E**), a synthetic SylA derivative that lacks the double bond required for covalent proteasome inhibition [20]. Inhibition assays at various SylA concentrations showed that proteasome inhibition was detectable at concentrations greater than 6.3 µM and that inhibition was always incomplete (**Figures 3E** and **3F**). These data are consistent with the observation that SylA preferentially inhibits two of the three catalytic subunits of the Arabidopsis proteasome, suggesting that the remaining signal was caused by the β1 catalytic subunit [20].

### SylA Blocks SA Signaling and SA-mediated Immunity in *N. benthamiana*

**Figure 4:**
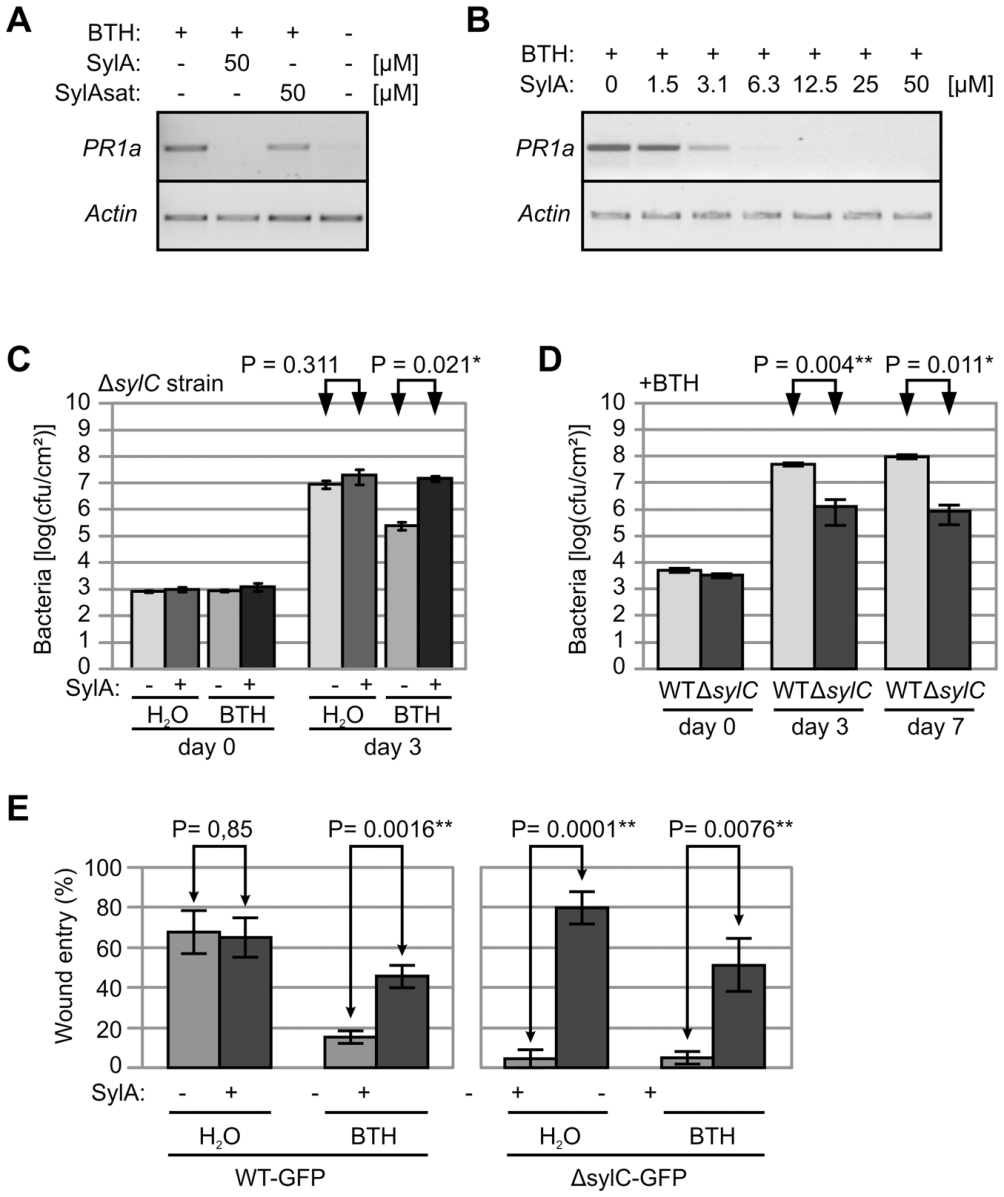
SylA blocks SA-mediated immunity. (**A**) SA signaling is blocked by SylA but not SylAsat. *N. benthamiana* leaves were infiltrated with 50 µM SylA or SylAsat and sprayed with 300 µM BTH. RNA was isolated from infiltrated tissues 6 h after BTH treatment and used as a template for semi-quantitative RT-PCR with gene-specific primers for *PR1a* and *Actin*. (**B**) Dose-dependent inhibition of BTH-induced *NbPR1a* expression by SylA. Leaves were infiltrated with various concentrations of SylA and sprayed 6 h later with 300 µM BTH. RNA was isolated from infiltrated tissues 6 h after BTH treatment and used as a template for semi-quantitative RT-PCR with gene-specific primers for *PR1a* (30 cycles) and *Actin* (24 cycles). (**C**) SylA blocks BTH-induced acquired resistance. *N. benthamiana* leaves were infiltrated with or without SylA and immediately sprayed with BTH. The Δ*sylC* mutant bacteria were infiltrated with 2×10^5^ bacteria/mL 6 h after SylA/BTH treatment, and bacterial populations were determined at 0 and 3 d after inoculation. Error bars represent SEM of four independent bacterial counts. This experiment was repeated three times with similar results. (**D**) SylA-producing bacteria grow better in BTH-treated tissues than Δ*sylC* bacteria. WT and Δ*sylC* bacteria were infiltrated into *N. benthamiana* leaves at 2×10^5^ bacteria/mL, and plants were sprayed with 300 µM BTH 6 h later. Bacterial populations were determined at 0, 3, and 7 dpi. Error bars represent SEM of four independent bacterial counts. This experiment was repeated two times with similar results. (**E**) SylA promotes wound entry in BTH-treated tissue. Leaves of *N. benthamiana* plants were infiltrated with 50 µM SylA or 0.025% DMSO. After 1 h, the infiltrated area was wound-inoculated with GFP-expressing WT or Δ*sylC* bacteria and sprayed with 300 µM BTH or water. Wound entry was scored at 5 dpi by fluorescence microscopy. Error bars represent SEM of four independent experiments. P-values determined using the Student's *t*-test are indicated.

Since wound entry might be associated with the suppression of acquired resistance in adjacent tissues, we tested whether SylA could inhibit SA signaling, a key regulator of acquired resistance in tobacco [26]. Treatment of *N. benthamiana* plants with the SA analog benzothiadiazole (BTH, 300 µM [27]) resulted in the transcriptional activation of the SA-responsive *PR1a* gene within 6 h (**Figure 4A**). However, this transcriptional activation of *PR1a* was blocked when tissues were preinfiltrated with 50 µM SylA, but not SylAsat (**Figure 4A**), indicating that SylA blocked SA signaling by proteasome inhibition. A SylA dilution series showed that suppression of SA signaling occurred at 3.1 µM SylA, but complete inhibition of SA signaling required concentrations greater than 12.5 µM SylA (**Figure 4B**).

To determine if SylA could also block immunity mediated by SA signaling, we infected BTH- and water-treated plants with Δ*sylC* bacteria in the absence or presence of SylA. In the absence of SylA, BTH treatment caused reduced bacterial growth of the Δ*sylC* strain compared to the control (**Figure 4C**). However, SylA treatment increased growth of Δ*sylC* bacteria in BTH-treated tissue to the same level as the control (**Figure 4C**). These data demonstrate that exogenous SylA can complement bacterial growth of the Δ*sylC* strain by suppressing SA-mediated immunity.

The above experiments indicate that SylA-producing WT bacteria will grow better during SA signaling than SylA-deficient Δ*sylC* bacteria. To test this hypothesis, we inoculated BTH-treated plants with WT and Δ*sylC* bacteria and measured bacterial growth. These assays demonstrated a statistically significant growth reduction of the Δ*sylC* mutant bacteria compared to WT bacteria (**Figure 4D**). Thus, during SA signaling, SylA-producing WT bacteria grow better than SylA-deficient Δ*sylC* bacteria, in contrast to naive plants where both strains grow equally well (**Figure 1G**).

To test whether SA signaling suppresses wound entry, we inoculated plants with WT-GFP and Δsyl*C*-GFP 1 d after spraying with water or BTH. BTH treatment significantly reduced the frequency of wound entry by WT bacteria (**Figure 4E**). Thus, activated SA signaling for 1 d was sufficient to suppress wound entry, even of SylA-producing WT bacteria. However, addition of exogenous SylA before BTH treatment promoted wound entry of both WT-GFP and Δ*sylC*-GFP bacteria in BTH-treated tissue (**Figure 4E**). Thus, SylA can promote wound entry by inhibiting SA signalling downstream of SA.

### SylA Deficiency Triggers Early Host Cell Death and Immune Responses

**Figure 5:**
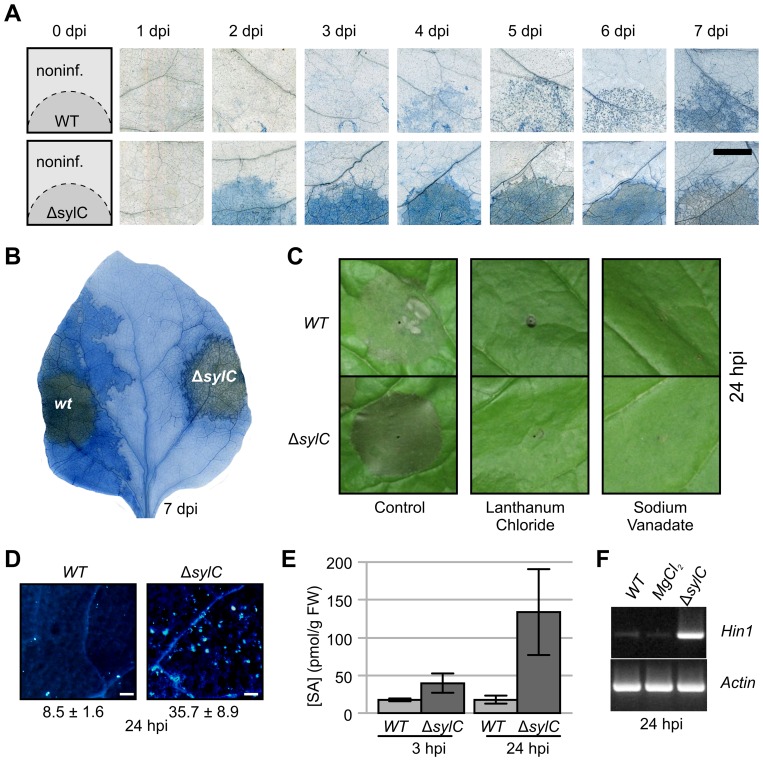
SylA-deficiency triggers immune responses at primary infection sites. (**A**) Time course of cell death induced by WT and Δ*sylC* strains. Leaves were stained with trypan blue at various days after infiltration. Scale bar, 10 mm. (**B**) Cell death spreads from zones infected with WT but not Δ*sylC* bacteria. Leaves were infiltrated and stained with trypan blue at 7 dpi. (**C**) Cell death induced by Δ*sylC* is blocked by the calcium transport inhibitor lanthanum chloride and the ATPase inhibitor sodium vanadate. Leaves were co-infiltrated with 1×10^8^ bacteria/mL with 50 µM lanthanum chloride or 1 µM sodium vanadate, and pictures were taken at 24 hpi. (**D**) Early host cell death induced by SylA-deficient Δ*sylC* bacteria is preceded by callose deposition. Leaves were stained for callose at 24 hpi, examined by fluorescence microscopy, and depicted with equal settings. The callose spots per 0.56 mm^2^ were quantified and printed below the picture with the SEM (n = 3). Scale bar, 0.1 mm. (**E**) Early host cell death induced by SylA-deficient Δ*sylC* bacteria is preceded by SA accumulation. *N. benthamiana* plants were infiltrated with 10^5^ bacteria/mL of WT and Δ*sylC* bacteria, and SA concentrations were measured at 3 and 24 hpi. Error bars represent SEM of three technical replicates. Student's t-test: P = 0.21 (3 hpi) and P = 0.096 (24 hpi). The experiment was repeated twice with similar results. (**F**) Early host cell death induced by SylA-deficient Δ*sylC* bacteria is preceded by upregulated transcript levels of the hypersensitive cell death marker *Hin1*. Semi-quantitative RT-PCR was performed on mRNA isolated at 24 hpi. (**A–F**) Bacteria were infiltrated with 2×10^5^ bacteria/mL into mature leaves of *N. benthamiana* and analyzed at 24 hpi unless stated otherwise.

While performing the infiltration assays, we noticed that Δ*sylC* bacteria triggered early host cell death upon infiltration (**Figure 5A**). Cell death upon Δ*sylC* inoculation occurred at 2 dpi, whereas WT bacteria caused host cell death usually after 5 dpi (**Figure 5A**). At later time points, late cell death developing upon infiltration of the WT strain often spread into tissues surrounding the primary infection site, whereas early cell death induced by the Δ*sylC* strain remained confined to the infiltrated region (**Figure 5B**).

To test whether Δ*sylC*-induced early host cell death is a form of programmed cell death reminiscent of the hypersensitive response (HR), we co-inoculated leaves with lanthanum chloride and sodium vanadate, two chemicals that can prevent programmed cell death by blocking transport of calcium ions and ATPase activity, respectively [28]. Importantly, Δ*sylC*-induced cell death was blocked both by lanthanum chloride and sodium vanadate (**Figure 5C**), indicating that early host cell death is a program that can be blocked.

Early host cell death by Δ*sylC* bacteria was preceded at 1 dpi by callose deposition (**Figure 5D**), increased SA levels (**Figure 5E**), and transcriptional activation of the HR-marker *Hin1* (**Figure 5F**). These data demonstrate that the Δ*sylC* strain triggered early cell death associated with typical HR-like responses. This is remarkable, since the growth of Δ*sylC* bacteria was indistinguishable from that of WT bacteria during these assays (**Figure 1G**).

### Δ*sylC-*induced Local Responses Are Weak when Compared to ETI/NHR Responses

**Figure 6:**
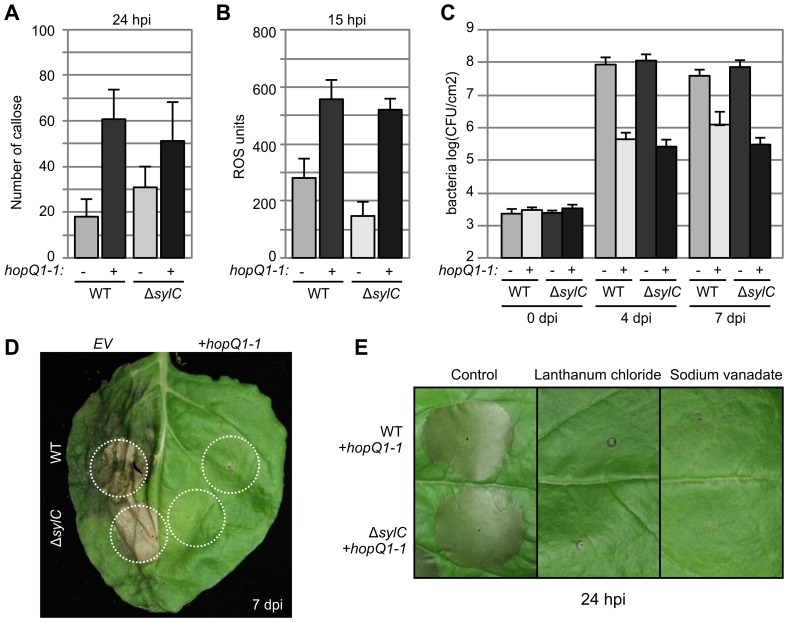
Δ*sylC*-induced immune responses are weak compared to ETI/NHR responses. (**A**) Increased callose deposition by both WT and Δ*sylC* strains expressing the HopQ1-1 effector. Leaves were infiltrated with 2×10^7^ bacteria/mL, and fluorescent callose spots were quantified per 0.56 mm^2^ after aniline blue staining at 24 hpi. Error bars indicate SEM of 20 technical replicates. These experiments were repeated three times with similar results. (**B**) Increased oxidative burst by both WT and Δ*sylC* strains expressing the HopQ1-1 effector. Leaves were infiltrated with 2×10^8^ bacterial cells/mL, and reactive oxygen species (ROS) were measured at 15 hpi in leaf discs floating on 200 µM MOPS containing L-012 for 20 min. The reduced ROS level of Δ*sylC*(EV)-infiltrated tissue compared to WT(EV)-infiltrated tissue was due to the late time point and the fact that ROS levels are transient. Error bars indicate SEM of eight technical replicates. These experiments were repeated three times with similar results. (**C**) Reduced bacterial growth of both WT and Δ*sylC* bacteria expressing the HopQ1-1 effector. Leaves were infiltrated with 2×10^5^ bacterial cells/mL in *N. benthamiana*, and bacterial growth was measured at 0, 4, and 7 dpi. Error bars represent standard deviation of four biological replicates. The experiment was repeated four times with similar results. (**D**) HopQ1-1-expressing WT and Δ*sylC* bacteria do not cause symptoms when infiltrated at low densities. Leaves were infiltrated at 2×10^5^ bacterial cells/mL, and pictures were taken at 7 dpi. (**E**) Cell death triggered by HopQ1-1-expressing WT and Δ*sylC* bacteria is blocked by the calcium transport inhibitor lanthanum chloride and the ATPase inhibitor sodium vanadate. Leaves were co-infiltrated with 1×10^8^ bacteria/mL with 50 µM lanthanum chloride or 1 µM sodium vanadate, and pictures were taken at 24 hpi.

To further investigate why immune responses induced by the Δ*sylC* strain did not affect bacterial growth, we transformed WT and Δ*sylC* strains with *hopQ1-1* of PtoDC3000, a type III effector that triggers nonhost resistance (NHR) in *N. benthamiana* [29]. Immune responses triggered by strains carrying *hopQ1-1* were compared to strains containing the empty vector (EV). Importantly, the presence of the HopQ1-1 effector increased callose deposition (**Figure 6A**) and reactive oxygen species in the Δ*sylC* strain (**Figure 6B**), indicating that SylA deficiency of the Δ*sylC* strain induces immune responses that are similar but much weaker compared to the ETI/NHR responses induced by HopQ1-1 [21]. Interestingly, HopQ1-1 also induced strong immune responses in the WT strain (**Figures 6A** and **6B**), indicating that SylA does not suppress HopQ1-1-induced responses.

Importantly, the presence of the HopQ1-1 effector in WT and Δ*sylC* strains was associated with strongly reduced bacterial growth upon infiltration, as expected for a strong ETI response (**Figure 6C**). Leaf tissue infiltrated with HopQ1-1-expressing WT and Δ*sylC* bacteria did not exhibit disease symptoms at low cell densities (**Figure 6D**), in agreement with the reduced bacterial growth. In contrast, early host cell death, induced by Δ*sylC*(EV), occurred shortly after infection but stayed confined to the infiltrated area. Cell death induced by WT(EV) occurred later and expanded beyond the infiltrated area (**Figure 6D**). However, when infiltrated at high densities, HopQ1-1-expressing WT and Δ*sylC* bacteria triggered cell death that could be blocked by lanthanum chloride and sodium vanadate (**Figure 6E**). Taken together, these data indicate that SylA-deficiency leads to an ETI-like immune response that is too weak to suppress bacterial growth.

### SylA Diffuses and Inhibits the Proteasome in Adjacent Tissue

**Figure 7:**
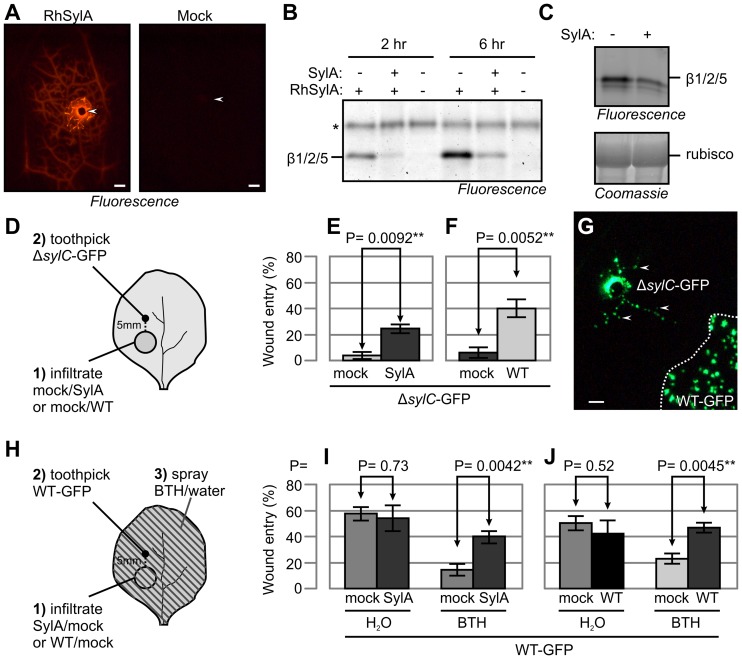
SylA diffuses and suppresses SA-mediated immunity in adjacent tissue. (**A**) RhSylA spread through the vasculature. A 1-µl aliquot of 2 mM RhSylA or 0.1% DMSO (mock) was applied at a wound site, and a fluorescence image was taken 2 h later. Scale bar, 1 mm. Arrowheads indicate wound inoculation sites. (**B**) RhSylA targets the proteasome in adjacent tissue. A 1-µl aliquot of 2 mM SylA was applied to an inoculation site and preincubated for 30 min. Subsequently, 1 µl of 2 mM RhSylA was added and incubated for another 2 h or 6 h. Proteins were extracted from tissue at 1–10 mm from the application site, and labeled proteins were detected by fluorescence scanning. *, background signal. (**C**) SylA targets the proteasome in adjacent tissue. A 1-µl aliquot of 1 mM SylA was applied at a wound site and incubated for 4 h. The application site was removed, extracts from adjacent tissues were labeled with MVB072, and fluorescently labeled proteins were detected. (**D**) Procedure for assaying wound entry by Δ*sylC*-GFP bacteria in adjacent tissue. Leaves of WT *N. benthamiana* were infiltrated with 50 µM SylA and 0.25% DMSO (E), 10^5^ WT bacteria or water (F), and the infiltrated region was marked. After 1 h (for SylA infiltration) or 1 d (for bacterial infiltration), Δ*sylC*-GFP bacteria were inoculated at a site 5 mm outside the infiltrated area. Wound entry was scored 5 d later by fluorescence microscopy. (**E**) SylA promotes wound entry by Δ*sylC*-GFP bacteria at a distance from the infiltrated region. (**F**) WT bacteria promotes wound entry at a distance from the infiltrated region. (**G**) Representative example of distant colonization of Δ*sylC*-GFP bacteria when inoculated next to areas infiltrated with WT-GFP bacteria. WT-GFP bacteria were infiltrated at 10^5^ bacterial cells/mL (lower right, bordered by dashed line). One day later, Δ*sylC*-GFP bacteria were inoculated at 5 mm from the infiltrated region. The picture was taken 5 d later. WT-GFP bacteria did not spread outside the infiltrated zone, but their presence promoted wound entry by Δ*sylC*-GFP in adjacent tissue. Arrowheads indicate colonies of Δ*sylC*-GFP in tissues adjacent to the wound inoculation site. (**H**) Procedure for assaying adjacent colonization by WT-GFP bacteria in adjacent tissue. Leaves of WT *N. benthamiana* were infiltrated with 50 µM SylA, 0.25% DMSO (H), 10^5^ WT bacteria or water (I), and the infiltrated region was marked. After 1 h (for SylA infiltration) or 1 d (for WT bacteria infiltration), WT-GFP bacteria were inoculated at 5 mm outside the infiltrated area and the plant was sprayed with 300 µM BTH or water. Wound entry was scored 5 d later by fluorescence microscopy. (**I**) SylA promotes wound entry in BTH-treated tissue at a distance from the infiltrated region. (**J**) SylA-producing WT bacteria promotes wound entry in BTH-treated tissue at a distance from the infiltrated region. (**E, F, I, J**) Error bars represent SEM of four independent biological replicates, each with 12 wound inoculations. P-values determined using the Student's *t*-test are indicated.

Since SylA is a small molecule that facilitates wound entry, we tested whether SylA could act at a distance from the primary inoculation site. Therefore, we inoculated RhSylA and monitored rhodamine-based fluorescence by fluorescence microscopy. These studies demonstrated that RhSylA moved through the vasculature up to 1 cm from the inoculation site within 2 h (**Figure 7A**). To test whether RhSylA also targeted the proteasome in these adjacent tissues, we extracted proteins from adjacent tissues and analyzed labeling by protein gel electrophoresis. These experiments demonstrated that RhSylA labeled the proteasome in adjacent tissue (**Figure 7B**). Co-inoculation of RhSylA with unlabeled SylA suppressed labeling, indicating that SylA itself could also move to adjacent tissue (**Figure 7B**). To confirm that SylA itself could inhibit the proteasome in adjacent tissues, we inoculated SylA locally and monitored proteasome activity in extracts of adjacent tissue using MVB072 labeling. The suppression of MVB072 labeling upon inoculation with SylA (**Figure 7C**) demonstrate that SylA inhibits the proteasome in adjacent tissues.

### SylA Promotes Wound Entry by Suppressing SA-mediated Immunity at a Distance

To investigate whether SylA also promotes colonization at adjacent sites, we infiltrated 50 µM SylA or a mock control into *N. benthamiana* leaves and inoculated Δ*sylC*-GFP bacteria at 5 mm outside the infiltrated region (**Figure 7D**). The Δ*sylC*-GFP bacteria colonized adjacent tissue significantly more frequently in tissue next to SylA-infiltrated zones compared to zones infiltrated with the mock control (**Figure 7E**), demonstrating that SylA promotes wound entry in adjacent tissues. To investigate whether SylA-producing WT bacteria also promoted wound entry by Δ*sylC* bacteria in adjacent tissues, we infiltrated WT bacteria and inoculated Δ*sylC*-GFP bacteria at 5 mm from the infiltrated area 1 d after infiltration. The presence of a nearby WT-infiltrated region significantly enhanced wound entry of the Δ*sylC*-GFP strain, compared to a mock-infiltrated region (**Figure 7F**).

To determine whether WT bacteria spread outside the infiltrated area, we repeated the assay by infiltrating WT-GFP bacteria followed by wound inoculation of Δ*sylC*-GFP bacteria in adjacent tissue. The empty space between the marked infiltration zone and the bacterial colonies originating from the wound inoculation site distinguished the WT-GFP from the Δ*sylC*-GFP bacteria (**Figure 7G**). Colonization of adjacent tissues by infiltrated WT-GFP bacteria did not occur at 6 dpi, whereas the Δ*sylC*-GFP bacteria were already colonizing adjacent tissues from wounding sites (**Figure 7G**). Thus, WT bacteria promote wound entry of Δ*sylC*-GFP bacteria at adjacent sites, presumably by producing diffusing molecules, rather than being present themselves.

To demonstrate that SylA suppresses SA signaling in adjacent tissues, we (1) infiltrated leaves with SylA or mock control; (2) wound inoculated WT-GFP next to the infiltrated area; and (3) sprayed plants with BTH to induce SA signaling (**Figure 7H**). BTH treatment resulted in a strong suppression of wound entry by WT-GFP bacteria compared to the water-treated control (**Figure 7I**), confirming that SA signaling suppresses wound entry even of WT bacteria (**Figure 4E**). Importantly, exogenous SylA infiltrated at a distance from the inoculated area significantly increased the frequency of wound entry by WT-GFP bacteria in BTH-treated tissue (**Figure 7I**). To test whether SylA-producing WT bacteria also suppressed SA-mediated immunity in the vasculature of adjacent tissues, we wound inoculated WT-GFP next to a zone infiltrated with WT bacteria 1 d after infiltration and sprayed the plants with BTH. BTH treatment suppressed wound entry of WT-GFP bacteria, but the presence of a nearby WT-infiltrated zone significantly enhanced wound entry of WT-GFP bacteria in BTH-treated tissues (**Figure 7J**). These data demonstrate that SylA-producing bacteria suppress SA responses in the vasculature of adjacent tissues.

### A Second Layer of Immunity: *NahG* Blocks Immunity in Adjacent Tissues but Not Escape from Primary Infection Sites

**Figure 8:**
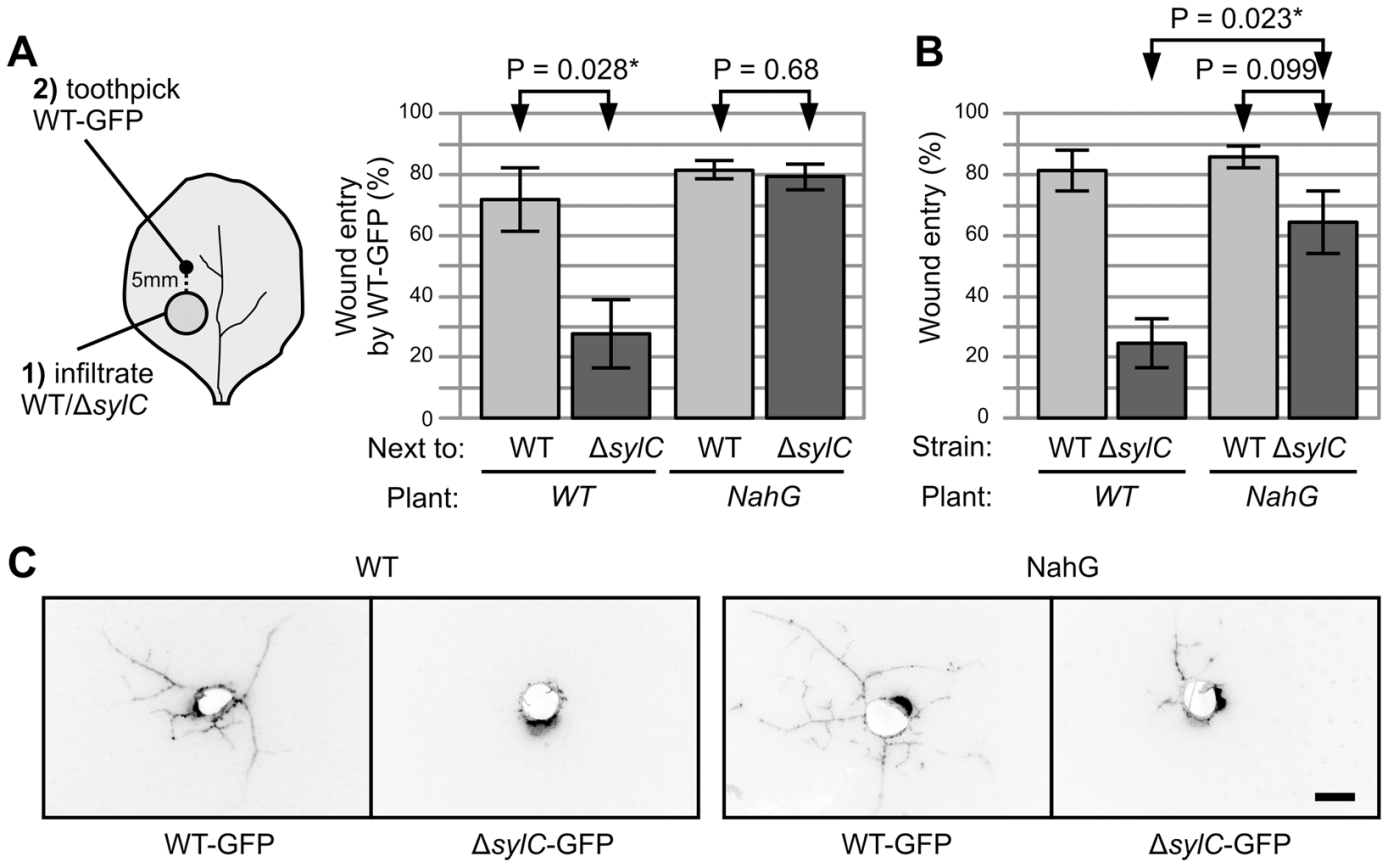
*NahG* blocks immunity in adjacent tissues and only partially promotes wound entry by *ΔsylC* bacteria. (**A**) Reduced wound entry by WT bacteria when inoculated next to Δ*sylC*-infiltrated regions is absent in *NahG* plants. Leaves of WT and *NahG*-transgenic *N. benthamiana* plants were infiltrated with WT or Δ*sylC* bacteria, and GFP-expressing WT PsyB728a bacteria were inoculated 1 d later at 0.5 cm from the border of the infiltrated region. Wound entry was monitored 5 d later by fluorescence microscopy. Error bars represent SEM of four independent experiments, each with 12 wound inoculations. P-values determined using the Student's t-test are indicated. (**B–C**) The Δ*sylC* mutant can colonize adjacent tissues in *NahG*-transgenic plants, though less than WT bacteria. WT and Δ*sylC* mutant bacteria were inoculated in WT and *NahG*-transgenic *N. benthamiana* plants, and wound entry was scored after 5 d by fluorescence microscopy. Error bars represent SEM of four independent experiments, each with 12 inoculations. P-values determined using the Student's t-test are indicated. (**C**) Representative pictures of colonization by WT or Δ*sylC* bacteria at 5 dpi in WT or *NahG*-transgenic plants. Fluorescence pictures were converted into inverted greyscale for better visibility. Scale bar, 1 mm.

The accumulation of SA during infection with the Δ*sylC* strain suggested that adjacent tissues might have acquired resistance. To test whether tissues surrounding Δ*sylC*-infected areas also mounted immunity, we inoculated WT bacteria next to regions that were preinfiltrated with WT or Δ*sylC* bacteria (**Figure 8A**, left). When inoculated next to Δ*sylC*-infiltrated regions, WT bacteria were unable to spread (**Figure 8A**), indicating that Δ*sylC*-infiltrated regions triggered acquired resistance in adjacent tissue. We performed the same experiment on *NahG*-transgenic *N. benthamiana* plants which cannot accumulate SA because *NahG* expresses a bacterial salicylate hydroxylase that converts SA into catechol [30]. When WT bacteria were inoculated next to Δ*sylC*-infiltrated regions in *NahG*-transgenic plants, wound entry was observed with the same frequency as when inoculated nest to regions infiltrated with WT bacteria (**Figure 8A**). These data show that *NahG* blocks immunity in adjacent tissues triggered by Δ*sylC* mutant bacteria.

To test whether blocking immune responses in adjacent tissues was sufficient for wound entry by SylA-deficient bacteria, Δ*sylC*-GFP bacteria were inoculated into *NahG*-transgenic plants. Importantly, we observed more frequent wound entry by Δ*sylC* bacteria from inoculation sites on *NahG* plants compared to WT plants (**Figure 8B**). However, the frequency of wound entry by Δ*sylC* bacteria was still less than that of WT bacteria. This difference was also evident from the colonization pattern upon wound entry: the number of veins along which Δ*sylC*-GFP bacteria colonized was significantly less compared to WT-GFP in *NahG* plants (**Figure 8C**). These data indicate that colonization from wound sites is suppressed by two mechanisms: an immune response in adjacent tissues, which is absent in *NahG* plants, and immune responses at the primary infection site that are not suppressed by *NahG*.

### Local Control of Wound Entry Is Associated with Reduced Motility

**Figure 9:**
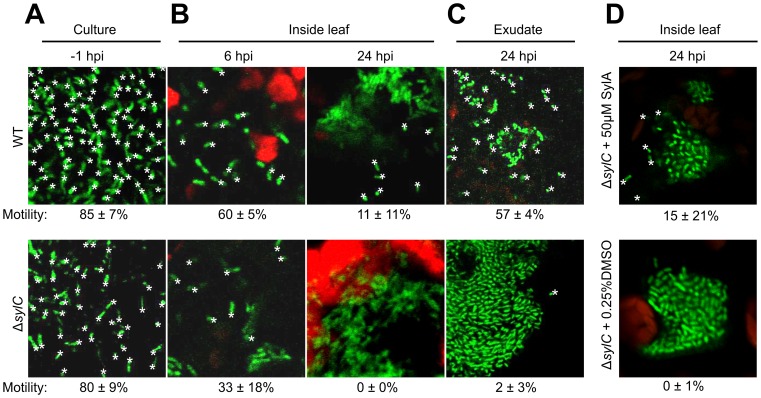
Δ*sylC* bacteria loose motility during infection. GFP-expressing WT and Δ*sylC* bacteria were monitored by confocal microscopy before infiltration (A), after infiltration (B), in the exudate of infected leaves (C), and after co-infiltration with or without SylA (D). All motile bacteria are marked with a star. The percentages of motile bacteria over a 2 s timeframe are indicated on the bottom, with standard deviations for n = 5. See supplemental data for the movies and details. Bacteria are 1–2 µm long. These results are representative of three independent experiments. *, motile bacterium.

Our data indicate that, in addition to SA-dependent immune responses in adjacent tissues, local immune responses also suppress the escape of SylA-deficient bacteria from primary infection sites. To determine whether Δ*sylC*-GFP bacteria have reduced motility at the primary infection site, WT-GFP and Δ*sylC*-GFP bacteria were monitored by confocal microscopy at different time points during the wound infection assays. The bacterial motility was indistinguishable between WT-GFP and Δ*sylC*-GFP bacteria when grown in NYG liquid medium (**Figure 9A** and **Movie S1** and **S2**) and 6 h after infiltration (**Figure 9B** and **Movie S3** and **S4**). At later stages, colonies developed in the leaf for both WT-GFP and Δ*sylC*-GFP bacteria. Bacteria inside these colonies were not motile (**Figure 9B** and **Movies S5** and **S6**). However, bacteria at the edge of WT-GFP colonies were motile (**Figure 9B** and **Movie S5**), but bacteria at the edge of Δ*sylC*-GFP colonies were not (**Figure 9B** and **Movie S6**). Furthermore, WT-GFP bacteria in exudates that leaked from a cut through an infected leaf were motile (**Figure 9C** and **Movie S7**), but Δ*sylC* bacteria only gained motility at the edge of the exudate and seemed to be embedded in a glue-like matrix (**Figure 9C** and **Movie S8**). The reduced motility of Δ*sylC* bacteria could be complemented chemically by co-infiltrating exogenous 50 µM SylA but not the mock control (**Figure 9D** and **Movies S9** and **S10**), confirming that the reduced motility of the Δ*sylC* mutant is caused by the absence of SylA. In conclusion, bacterial motility was identical during the initial stages of infection and was reduced for Δ*sylC*-GFP bacteria during the later stages of infection.

## Discussion

**Figure 10:**
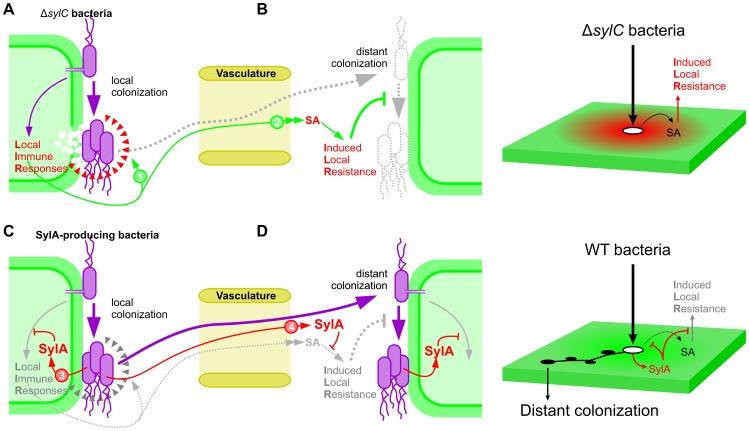
Model of SylA action. (**A–B**) SylA-deficient Δ*sylC* bacteria trigger local immune responses, resulting in both local HR-like cell death and immune responses (red triangles) and induced local resistance (ILR) in adjacent tissue, which is dependent on SA signaling. Wound entry is prevented by both local (1) and adjacent (2) immune responses. Only a few Δ*sylC* bacteria can escape (dashed line) to establish colonies in adjacent tissues in the absence of SA signaling. (**C–D**) SylA-producing WT bacteria secrete SylA, which prevents immune responses at the primary infection site (3). In addition, SylA diffuses over a distance and prevents acquired resistance induced by SA signaling (4). Consequently, SylA-producing bacteria can escape from primary infection sites and colonize adjacent tissue.

This work uncovered a key role for SylA in facilitating wound entry by suppressing immune responses, both locally and in adjacent tissue. A model for the dual role of SylA is summarized in **Figure 10** and is discussed below. Our data indicate that the role of SylA as a virulence factor depends on the assay. SylA is a virulence factor in the classical sense, since the SylA-deficient mutant causes less symptoms on bean plants upon spray inoculation [18]. SylA is also a virulence factor with respect to promoting bacterial growth, since the *ΔsylC* mutant grew less compared to the WT strain in the first hours after spray inoculation, which can be explained by the fact that SylA suppresses stomatal closure [19]. However, we did not detect a statistically significant virulence role for SylA on bacterial growth upon infiltration, despite the different conditions tested (**Figure 1G** and **S1**). In contrast, we demonstrated that SylA promoted bacterial growth in BTH-treated plants (**Figure 4CD**) and wound entry (**Figure 1A–E**), associated with a 63-fold increased bacterial population level (**Figure 1F**). We believe that this phenotype is important for the biology of PsyB782a and PsyB301D, since both strains are known to infect bean leaves and pear blossoms via wounds caused by wind or frost, respectively [7], [8].

### 1. SylA Deficiency Triggers Local Immune Responses without Affecting Bacterial Populations

SylA-deficient PsyB728a strains triggered local immune responses, including early host cell death and the accumulation of SA, callose, and *Hin1* transcripts (**Figure 5**). The Δ*sylC*-induced early host cell death could be prevented with calcium channel or ATPase inhibitors (**Figure 5C**), indicating that this is a form of programmed cell death. The Δ*sylC*-induced immune responses were relatively weak compared to NHR/ETI responses induced by the presence of HopQ1-1 (**Figure 6**), which explains why SylA-deficient bacteria grew equally well locally compared to WT bacteria (**Figure 1G**). PsyB728a carries several type III effectors that can trigger HR and other ETI responses, resulting in reduced bacterial growth in *N. benthamiana* [14]. PsyB728a is also likely to trigger PTI by flagellin and other elicitors [13]. The identification of the factor(s) that trigger immune responses in the absence of SylA was not the scope of this study but is important to classify the response triggered by the Δ*sylC* strain.

The observation that the SylA-deficient Δ*sylC* mutant grew similarly to WT bacteria upon infiltration (**Figures 1G** and **S1**), demonstrated the striking ability of PsyB728a to survive in dead host tissue, despite the widely accepted view that the HR and immune responses suppress pathogen survival. The survival of *P. syringae* in tissue undergoing HR and immune responses is not unprecedented. Unaffected bacterial populations in dying host tissue have also been observed in tobacco plants infiltrated with avirulent *P. syringae* pv. *maculicola* (*Pma*) M2 and pv. *tabaci* (*Pta*) [31]. The absence of cell death in *SGT1*-silenced *N. benthamiana* plants did not affect bacterial growth of PsyB728a and only moderately affected growth of PtoDC3000, *Pma*, and *Pta* [32]. High-throughput silencing in *N. benthamiana* has revealed many genes that suppress AvrPto/Pto-mediated HR when silenced, but only a few genes have an effect on bacterial growth [33]. These data indicate that *P. syringae* is able to survive and even grow in the presence of dead host tissue, despite the likely presence of toxic components generated by immune responses of the dying host. These data are consistent with the notorious survival of PsyB728a as an epiphyte, even during dry periods [3], [34].

### 2. SylA Deficiency Triggers SA Accumulation and Acquired Resistance in Surrounding Tissues

Inoculation of WT-GFP bacteria next to regions preinfiltrated with Δ*sylC* bacteria demonstrated that Δ*sylC* triggered immune responses in surrounding tissues that suppressed wound entry (**Figure 8A**). The Δ*sylC* bacteria triggered the accumulation of SA (**Figure 5E**), and SA is known to cause acquired resistance in tobacco [26]. These data demonstrated that Δ*sylC* bacteria triggered SA-dependent acquired resistance in adjacent tissue (ILR, induced local resistance) (**Figure 10A**). ILR is similar to the well-described systemic acquired resistance (SAR), which occurs in the entire plant and is also dependent on SA signaling [26]. The experiments with *NahG*-transgenic plants demonstrated that the suppression of wound entry occurred at two levels: an acquired response in surrounding tissues, absent in the *NahG* line (**Figure 8A**); and a local response confining the bacteria at the primary infection site, still mostly present in the *NahG* line (**Figure 8BC**). The suppression of acquired resistance in adjacent tissue in the *NahG* line might be due to degradation of SA or due to the accumulation of catechol, which can affect bacterial growth [35]–[36].

### 3. SylA Production Suppresses Local Immune Responses

In contrast to Δ*sylC* bacteria, WT bacteria did not trigger early cell death and prevented the early accumulation of callose, *Hin1* transcripts, and SA (**Figure 5**). Thus, SylA production suppressed local immune responses. The finding that local immune responses could be suppressed by inhibiting the proteasome is consistent with previous reports. Hatsugai and colleagues found that silencing of the β1 proteasome subunit suppressed AvrRpm1-induced HR [37]. Furthermore, proteasome inhibitors also have been shown to inhibit early cell death induced by *P. syringae* pv. *phaseolicola* [38] and pv. *tabaci* [31] in tobacco. Taken together, these studies implicate proteasome involvement in cell death and immunity triggered by *P. syringae*. Interestingly, the HopQ1-1-induced HR was not suppressed in SylA-producing WT bacteria (**Figure 6E**). These results either mean that SylA could not block all ETI responses or that the timing and/or concentration of SylA production was insufficient to prevent HopQ1-1-induced ETI.

The principle that proteasome inhibition can suppress early cell death might be counterintuitive, since proteasome inhibitors generally are assumed to cause cell death. However, proteasome inhibition can also promote cell survival or cell death, depending on the concentrations and potencies of the proteasome inhibitors used [39]. During PsyB728a infection, SylA intercepted a pro-death program, implying that SylA production was carefully timed and targeted by PsyB728a during infection to provide a sublethal dose. Proteasome inhibition also seems counterintuitive, since many effectors like AvrPtoB, HopM1, and coronatine depend on proteasome activity to execute degradation of their host targets [40]–[42]. The PsyB728a genome lacks biosynthesis genes for coronatine, but it contains AvrPtoB and HopM1 homologs [43]–[44]. Therefore, the SylA concentration and timing may be essential parameters that act in concert with the expression of other effectors. These data suggest that ETI suppression by SylA can be overruled if ETI is triggered stronger and/or faster, e.g. in the case of HopQ1-1.

### 4. SylA Diffuses and Creates a Zone of SA-insensitive Vasculature Tissue

Our data indicate that SylA diffuses through the vasculature and inhibits the host proteasome in adjacent tissues. Although analytical tools to detect SylA in plant tissues are not yet available, we showed that SylA suppressed proteasome activity in adjacent tissues (**Figure 7C**) and found that rhodamine-tagged SylA quickly moves through the vasculature (**Figure 7AB**). At this stage, it is unclear whether the SylA movement is apoplastic (through the xylem) or symplastic (through the phloem) and whether the transport is active or passive. Although SylA is produced by bacteria residing in the apoplast, SylA is also quickly taken up by host cells and may enter the symplastic transportation route [20].

Since SylA acts along the vasculature, we used vasculature-specific immunity assays to detect the suppression of SA signaling. For these assays, we used wound inoculations, which provide bacteria with direct access to the vasculature. Importantly, the presence of SylA, when supplemented at a distance, complemented wound entry by Δ*sylC*-GFP bacteria (**Figure 7E**). Also, SylA-producing WT bacteria promoted wound entry of Δ*sylC*-GFP bacteria in adjacent tissues (**Figure 7FG**), indicating that sufficient SylA was produced during infection to promote colonization from wounding sites.

SylA directly blocks SA signaling in *N. benthamiana*, which was demonstrated by blocking BTH-induced *PR1a* expression using SylA (**Figure 4A**). These data are consistent with the observation that SylA blocks SA signaling in Arabidopsis [19]. One possible molecular mechanism is that SylA prevents the degradation of phosphorylated NPR1 in the nucleus, which needs to be removed by the nuclear proteasome to allow continued transcriptional activation of NPR1-responsive genes [45]. The inhibition of SA signaling in the nucleus is consistent with the observation that SylA targets the nuclear compartment [20]. The subcellular targeting of SylA also explains why SylA blocks SA signaling at concentrations that are less than required for full proteasome inhibition in the total extracts. Thus, new assays are required to detect tissue-specific and subcellular proteasome inhibition during infection.

Importantly, we showed that SylA and SylA-producing bacteria blocked SA signaling in adjacent tissues, since BTH-mediated suppression of wound entry by WT-GFP bacteria was restricted when SylA or WT bacteria were preinfiltrated next to the infection sites (**Figure 7J**). Vasculature-specific infection assays were required, since SylA acted along the vasculature at a low dose. These experiments indicate that SylA-producing bacteria use effector diffusion to create a zone of SA-insensitive vasculature tissue around the infection site.

### Remote Control by Effector Diffusion

Our data indicate that SylA moves and inhibits the nuclear proteasome in host cells along the vasculature to create SA-insensitive tissue that was ready for subsequent colonization. Effector diffusion is a common strategy used by pathogens to control immune responses in adjacent tissues. Several fungal effectors that translocate to the host cytoplasm move to adjacent plant cells and are thought to prepare adjacent cells for infection [46]–[47]. These effectors typically move up to four adjacent cells (less than 0.1 mm). In contrast, small molecule effectors, such as SylA, can move over greater distances (several millimeters) and can reach a larger area surrounding the primary infection site. Although the SylA biosynthesis gene cluster so far only has been found in phylogroup II *P. syringae* strains, other strains produce other small molecule effectors that can interfere with SA signaling in adjacent tissues. A well-studied example is coronatine, which is produced by several *P. syringae* strains and also suppresses SA signaling, in this case by activating the jasmonate signaling cascade that results in the downregulation of the SA biosynthetic enzymes and upregulation of SA-converting enzymes [48]–[49]. *P. syringae* strains producing coronatine increase the susceptibility of noninoculated leaves, a phenomenon called systemic induced susceptibility (SIS) [50]. Coronatine is required and sufficient to induce SIS, and SIS is suppressed in *NahG*-transgenic plants. However, this increased susceptibility is only moderate (5-fold increased growth), and the biological relevance is unclear as it benefits other pathogens as well as the coronatine-producing strain. We focused on adjacent tissues because they are relevant for the wound entry phenotype. We found that SylA production is beneficial for SylA-producing strains as these strains can escape primary infection sites, and increase the bacterial population. These experiments indicate that control of adjacent tissue by effector diffusion may be a common strategy for *P. syringae* and that different strains use different toxins, in different hosts, and through different molecular mechanisms.

### Both Adjacent and Local Immune Responses Suppress Wound Entry

The absence of WT-like wound entry by Δ*sylC*-GFP bacteria in ILR-deficient *NahG* plants (**Figure 8**) indicated that also local responses prevent colonization from wound sites. The escape from local confinement in the ILR-deficient *NahG* plants occurred sporadically, resulting in only a few infected vasculatures where wound entry occurred (**Figure 8C**). Notably, time-resolved confocal microscopy demonstrated that Δ*sylC*-GFP bacteria were embedded in a rigid extracellular matrix (ECM) that seemed to immobilize the bacteria at the primary infection site (**Figure 9**). This rigid ECM was probably similar to the fibrillar ECM described in the 1980s for incompatible interactions with *P. syringae* bacteria [51]–[52]. The rigid ECM might have been derived from the bacteria or created by vacuolar content release during the HR. The proteasome is essential for fusion of the vacuolar membrane with the plasma membrane during the HR [37]. Therefore, inhibition by SylA could prevent vacuolar content release into the apoplast. However, a rigid ECM might not be the only possible mechanism of local confinement. Immune responses might also change the motility of the bacteria by affecting their metabolic state. The nature of the matrix, the metabolic status of the embedded bacteria, and how SylA prevents these events remain topics for future studies.

### Conclusion

In conclusion, our studies revealed that SylA facilitated wound entry of PsyB728a and PsyB301D by blocking immune responses in both local and adjacent tissues. Local immune responses did not affect bacterial population levels but contained the bacteria to the primary infection site. Immune responses in adjacent tissues were SA-dependent and suppressed colonization by bacteria that escaped confinement at the primary infection site. SylA suppressed this two-layered immune response by blocking immune responses in both local and adjacent tissues. Our data indicate that SylA diffuses through the vasculature and blocks SA signaling in adjacent tissues, creating an SA-insensitive zone of vasculature tissue that makes the tissue ready for subsequent colonization.

## Methods

### Generation of Transgenic Pseudomonas Strains

All plasmids and strains are summarized in Supplemental **Table S1**. The ORF encoding GFP was amplified from DNA isolated from GFP-transgenic *Pst*DC3000 [42] using primers 5′-tccccatgggtaaaggagaag-3′ and 5′-tccccatggttagagctctagttcatccatgccatg-3′ and cloned into pGEM-T (Promega), resulting in pFK69. The GFP ORF was amplified from pFK69 using primers 5′-atcgaagcttaggaggacagctatgggtaaaggagaaga-3′ (introducing a ribosome binding site, RBS) and 5′-gatgagctcctcgagtctagaatcgatctatttgtatagttcatccatgccatg-3′ and cloned into pBlueScript using HindIII and XbaI restriction sites, resulting in pFK74. The RBS-GFP cassette of pFK74 was cloned into pML123 [53] using BamHI and XbaI restriction sites, resulting in pFK78. *Pseudomonas syringae* strains were transformed with pFK78 by electroporation and selected on gentamycin (10 µg/mL). Several fluorescent colonies were used for infection and were found to behave similarly. *PacWT*-*GFP* was transformed with cosmid pPL3syl carrying *sylA-E* genes of *Psy*B301D-R [23] by triparental mating using a helper *E. coli* strain carrying pRK600 and selected with tetracyclin (10 µg/mL), gentamycin (10 µg/mL) and rifampicin (25 µg/mL) [23].

### Plant Growth and Infection Assays

*Nicotiana benthamiana* plants were grown under a 12 h light regime at 22°C and 60% relative humidity and used at 3–5 weeks old, before flowering, unless indicated otherwise. For infection by infiltration, bacteria were grown overnight in 10 mL NYG medium (5 g/L peptone, 5 g/L yeast extract, 2% glycerol), centrifuged and resuspended into 10 mM MgCl_2_. The OD_600_ was measured and bacteria were diluted to an OD_600_ of 0.0002 in 10 mM MgCl_2_. Bacteria were infiltrated into *N. benthamiana* leaves with a 1 mL syringe without needle. Leaves were examined at various time points. GFP fluorescence was detected by a Leica MZ16FA using the GFP filter. All experiments were done with similar acquisition settings.

For wound inoculation, the three youngest expanded leaves of 3–5 week-old, nonflowering *N. benthamiana* plant were selected. Bacteria were taken from fresh plates with a sterile toothpick and punched through the leaf using a new toothpick for every infection. In case of infection with controlled inoculum (**Figure 1F**), wound sites were immediately inoculated with 1 µL of a 10^7^ bacteria/mL; Usually two strains were compared in opposite leaf halves. Plants were incubated at 22°C without cover and pictures were taken at 5 dpi using stereo fluorescence microscopy. Pictures of every infection site were taken for blind scoring and verification. An example experiment is shown as **Figure S3**. The percentage of wound entry was calculated for at least 12 toothpicks by dividing the number of times that colonies appear in adjacent tissues by the number of times that colonization occurred at the toothpick inoculation site itself. Inoculation sites that did not show GFP fluorescence were rare and were not included in the count. The average and standard error of the mean (SEM) was calculated from four independent experiments.

Endophytic bacterial populations were determined by colony count assays from opposite leaf halves as described previously [54]. Briefly, leaves were surface-sterilized in 15% H_2_O_2_ for 5 min on a shaker at 200 rpm and washed with sterile water. Alternatively, leaves were sterilized by 70% ethanol for 5 min (Supplemental **Figure S1**). Leaf disks (13 mm diameter, unless otherwise indicated) from two infiltrated leaves were combined and ground in 1 mL 10 mM MgCl_2_ using metal beads. 20 µL droplets of a serial 10-fold dilution series were put on selective agar medium and colonies were counted after one day of incubation at 28°C. This procedure was repeated on at least three sets of infiltrated leaves for each experiment.

### SylA, BTH, ABPP and RT-PCR

Synthetic SylA, SylAsat, RhSylA and the epoxomicin-based proteasome probe MVB072 were described previously [20], [25], [55]. *N. benthamiana* leaves were infiltrated with various SylA concentrations. Similarly DMSO was used as a control. Plants were sprayed with 300 µM BTH (Actigard, Syngenta) or water. RNA was isolated at 6 or 12 h after BTH treatment using the Qiagen RNeasy mini kit, and cDNA was synthesized using Superscript II reverse transcriptase (Invitrogen) using oligo(dT) 20 primer (Invitrogen) according to the instructions of the manufacturer. cDNA was used as a template for PCR using primer pairs for *NbPR1:* *5′-*AATATCCCACTCTTGCCG-3′ and *5′-*CCTGGAGGATCATAGTTG-3′; *NbActin:* *5′-*TGGACTCTGGTGATGGTGTC-3′ and *5′-*CCTCCAATCCAAACACTGTA-3′; and *NbHin1*: 5′-GAGCCATGCCGGAATCCAAT-3′ and 5′-GCTACCAATCAAGATGGCATCTGG-3′. Activity-based protein profiling (ABPP) with MVB072 on SylA-treated tissues was performed as described previously [20]. Extracts from leaf discs were labelled with 1.6 µM MVB072 in 50 mM NaOAc pH 7 for 2 h and labelled proteins were detected from protein gels using the Typhoon 8600 scanner (Molecular Dynamics) with excitation and emission at 532 and 580 nm, respectively.

### Measurements of SA

SA concentrations were measured as described in Straus et al. [56]. Briefly, SA was extracted from 100 mg plant material in 1 ml chloroform/methanol/water (1∶2∶0.3) containing 160 pmol 2-hydroxybenzoic-3,4,5,6-d_4_ acid (SA-d_4_; Campro Scientific, http://www.campro.eu/) as internal standard. After shaking for 10 min at 70°C samples were centrifuged and re-extracted with 0.5 ml chloroform/methanol (1∶2). After phase separation through the addition of 0.5 ml H_2_O the polar extract was dried. Samples were acidified with 30 µl 10% trifluoroacetic acid (TFA) and extracted twice with 0.6 ml ethyl acetate/hexane (3∶1). Following evaporation of organic solvents, analytes were derivatized with 80 µl pyridine/*N*-methyl-*N*-(trimethylsilyl)trifluoroacetamide (1∶1) (Sigma) and 1 µl was injected into a gas chromatograph coupled to a mass spectrometer (GC-MS; Agilent, http://www.agilent.com/). Masses of SA-d_4_ (*m*/*z* 271) and SA (*m*/*z* 267) were detected by selected ion monitoring and quantified using the Chemstation software from Agilent.

### Callose Deposition Assays

Callose deposits were measured in leaves of *N. benthamiana* after infiltration with 2×10^7^ bacteria/mL. Leaf discs were excised 24 h after infiltration and evacuated in 95% ethyl alcohol at 37°C exchanging the ethyl alcohol every 2 h until cleared. Leaves were stained with the fluorescent dye aniline blue (0.01%) in a solution of 150 mM K_2_HPO_4_ (pH 9.5) for 30 m as previously described [57] then mounted on slides in 50% glycerol. The aniline blue-stained callose was visualized on a fluorescence microscope (Zeiss Axionplan 2, Carl Zeiss, Oberkochen, Germany), and the callose deposits were quantified using Image J (National Institutes of Health).

### Reactive Oxygen Species (ROS) Assays

*N. benthamiana* leaves were infiltrated with 2×10^8^ bacteria/mL. 15 h after infiltration leaf discs were taken with a 0.4 cm diameter cork borer and floated on 200 µL 10 mM morpholinepropanesulfonic acid (MOPS)/KOH (pH 7.4) containing 0.5 mM L-012 (Wako Pure Chemicals, Osaka, Japan) in a 96 well plate. Luminescence was measured at 20 min. using a Synergy 2 luminometer and quantified by Gen5 data analysis software (Biotek Instruments).

## Supporting Information

Figure S1 **Bacterial growth of WT and Δ** ***sylC*** **mutant PsyB728a upon infiltration.** GFP-expressing (A–B, C, G and H) or non-transgenic bacteria (D, E, F and I) were infiltrated with 2×10^5^ (A–G and I) or 2×10^4^ (H) bacteria/mL, and infected plants were kept at high (60–90%) relative humidity (RH) (A–F), 60% RH (F–H) or transferred at 2 dpi from high RH to 60% RH (I). Bacterial populations were determined at different days-post-inoculation (dpi). Leaves were surface-sterilized with hydrogen peroxide (A–C, G–I) or ethanol (D–E) before leaf extracts were generated, diluted and plated. Experiments were performed in Cologne (A–E, G–I) or Nebraska (F). (A–I) Independent leaves were taken for n independent counts, indicated at the bottom. All error bars represent SEM. Pairwise comparisons between WT and ΔsylC growth was calculated using the Student t-test. NA, not analyzed.
(JPG)

Figure S2 **Structures of chemicals used in this study.** Reactive groups (red), biotin (blue) and fluorescent reporter (yellow).
(JPG)

Figure S3 **Representative wound entry assay experiment.** WT-GFP and ΔsylC-GFP bacteria were toothpick-inoculated from a fresh plate into different leaves of different plants of *N. benthamiana*. Pictures were made at 5 dpi using stereo fluorescence microscopy using identical settings. The frequency of host entry at each wound inoculation sites was counted over 12 toothpick sites, as shown at the bottom. Scale bar, 1 mm.
(JPG)

Movie S1 **Bacterial motility of WT-GFP in hanging droplet assay.** *WT-GFP* bacteria were grown in NYG liquid medium and their motility was monitored by confocal microscopy by hanging droplet assay. Shown is the edge of the droplet for 200 seconds. The size of the movie frame corresponds to 512×512 µm.
(AVI)

Movie S2 **Bacterial motility of Δ** ***sylC-GFP*** **in hanging droplet assay.** Δ*sylC-GFP* bacteria were grown in NYG liquid medium and their motility was monitored by confocal microscopy by hanging droplet assay. Shown is the edge of the droplet for 200 seconds. The size of the movie frame corresponds to 512×512 µm.
(AVI)

Movie S3 **Bacterial motility of WT-GFP at 6 h after infiltration.** *WT-GFP* bacteria were infiltrated at 10^8^ bacteria/ml and their motility was monitored six hours later by confocal microscopy. The length of the movie is 200 seconds. The size of the movie frame corresponds to 512×512 µm.
(AVI)

Movie S4 **Bacterial motility of Δ** ***sylC-GFP*** **at 6 h after infiltration.** *ΔsylC-GFP* bacteria were infiltrated at 10^8^ bacteria/ml and their motility was monitored six hours later by confocal microscopy. The length of the movie is 200 seconds. The size of the movie frame corresponds to 512×512 µm.
(AVI)

Movie S5 **Bacterial motility of WT-GFP in colonies at 24 hpi.** Leaves were infiltrated with 10^5^ *WT-GFP* bacteria/mL and subjected to confocal microscopy at 24 hpi. Host cells are intact (middle and bottom) and carry normal-looking chloroplasts (red). Bacteria (green) are static in the colony (bottom), but are motile along the edge of the colony (top). The length of the movie is 200 seconds. The size of the movie frame corresponds to 512×512 µm.
(AVI)

Movie S6 **Bacterial motility of Δ** ***sylC-GFP*** **in colonies at 24 hpi.** Leaves were infiltrated with 10^5^ Δ*sylC-GFP* bacteria/mL and subjected to confocal microscopy at 24 hpi. Bacteria (green) are not motile and have occupied a collapsed host cell containing degenerating chloroplasts (red). The length of the movie is 200 seconds. The size of the movie frame corresponds to 512×512 µm.
(AVI)

Movie S7 **Bacterial motility of** ***WT-GFP*** **in colony exudates at 24 hpi.** Leaves were infiltrated with 10^5^ *WT-GFP* bacteria/mL, cut, and subjected to confocal microscopy at 24 hpi. GFP-expressing bacteria in the exudate that leaks from the leaf cut through infected tissue. The length of the movie is 100 seconds. The size of the movie frame corresponds to 512×512 µm.
(AVI)

Movie S8 **Bacterial motility of Δ** ***sylC-GFP*** **in colony exudates at 24 hpi.** Leaves were infiltrated with 10^5^ *ΔsylC-GFP* bacteria/mL, cut, and subjected to confocal microscopy at 24 hpi. GFP-expressing bacteria in the exudate that leaks from the leaf cut through infected tissue. The length of the movie is 100 seconds. The size of the movie frame corresponds to 512×512 µm.
(AVI)

Movie S9 **Bacterial motility at 24 hpi in** ***sylC-GFP*** **colonies with 50 µM SylA.** Leaves were infiltrated with 10^5^ ΔsylC-GFP bacteria/mL containing 50 µM SylA and bacterial colonies were imaged at 24 hpi by confocal microscopy. The length of the movie is 100 seconds. The size of the movie frame corresponds to 512×512 µm.
(GIF)

Movie S10 **Bacterial motility at 24 hpi in** ***sylC-GFP*** **colonies with 0.25% DMSO.** Leaves were infiltrated with 10^5^ ΔsylC-GFP bacteria/mL containing 0.25% DMSO and bacterial colonies were imaged at 24 hpi by confocal microscopy. The length of the movie is 100 seconds. The size of the movie frame corresponds to 512×512 µm.
(GIF)

Table S1 **Identification of MVB072-labeled proteins from** ***N. benthamiana*** **.**
(PDF)

Table S2 **Plasmids and strains used in this study.**
(PDF)
