# Supplementary figures and images for: Pseudomonas syringae pv. syringae Uses Proteasome Inhibitor Syringolin A to Colonize from Wound Infection Sites
Source: PLoS Pathog. 2013 Mar 28;9(3):e1003281. doi: 10.1371/journal.ppat.1003281 (PMC3610659; doi:10.1371/journal.ppat.1003281)

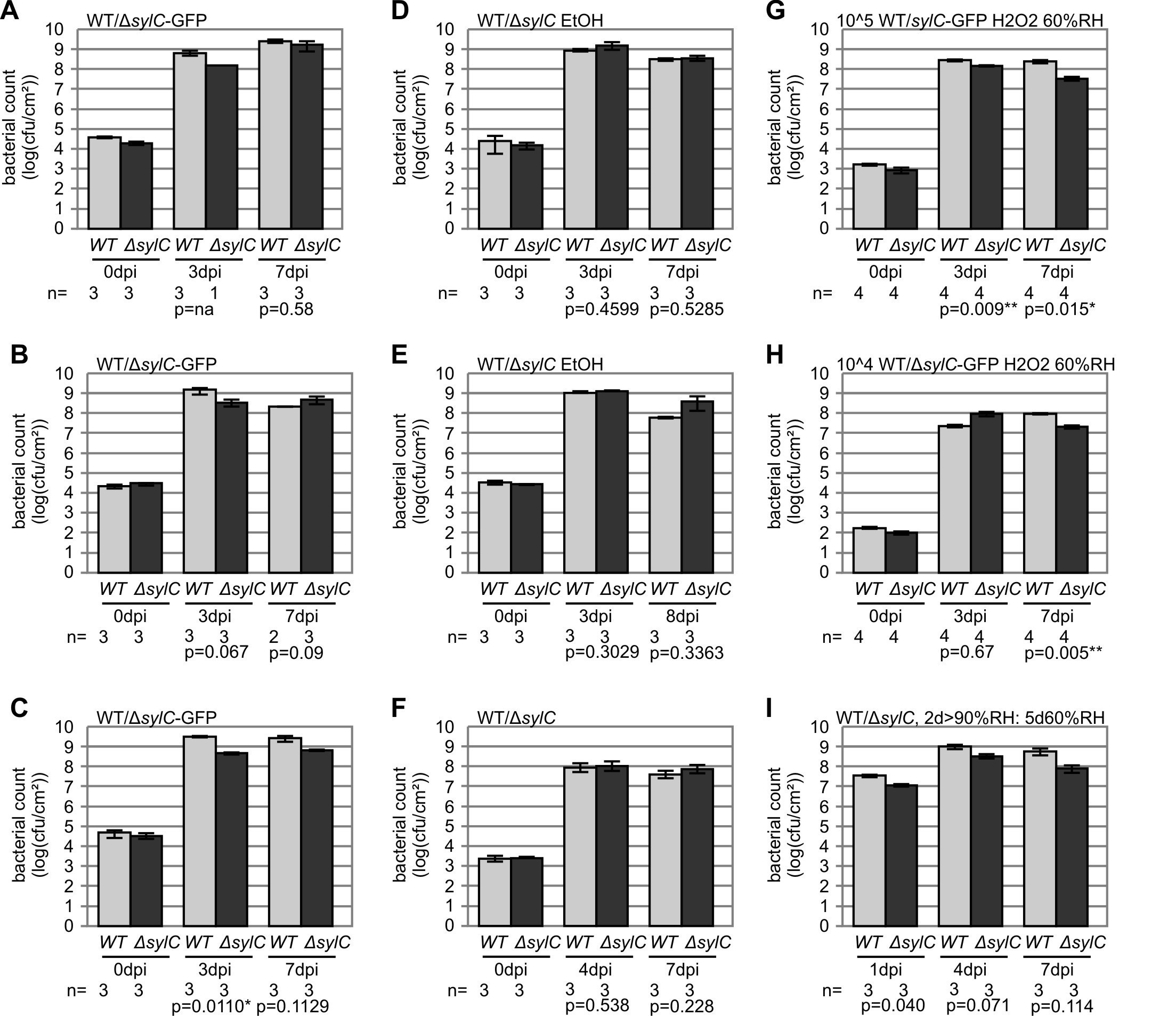

Supplement: Figure S1 — Bacterial growth of WT and Δ sylC mutant PsyB728a upon infiltration. GFP-expressing (A–B, C, G and H) or non-transgenic bacteria (D, E, F and I) were infiltrated with 2×10^5^ (A–G and I) or 2×10^4^ (H) bacteria/mL, and infected plants were kept at high (60–90%) relative humidity (RH) (A–F), 60% RH (F–H) or transferred at 2 dpi from high RH to 60% RH (I). Bacterial populations were determined at different days-post-inoculation (dpi). Leaves were surface-sterilized with hydrogen peroxide (A–C, G–I) or ethanol (D–E) before leaf extracts were generated, diluted and plated. Experiments were performed in Cologne (A–E, G–I) or Nebraska (F). (A–I) Independent leaves were taken for n independent counts, indicated at the bottom. All error bars represent SEM. Pairwise comparisons between WT and ΔsylC growth was calculated using the Student t-test. NA, not analyzed. (JPG) [file ppat.1003281.s001.jpg]

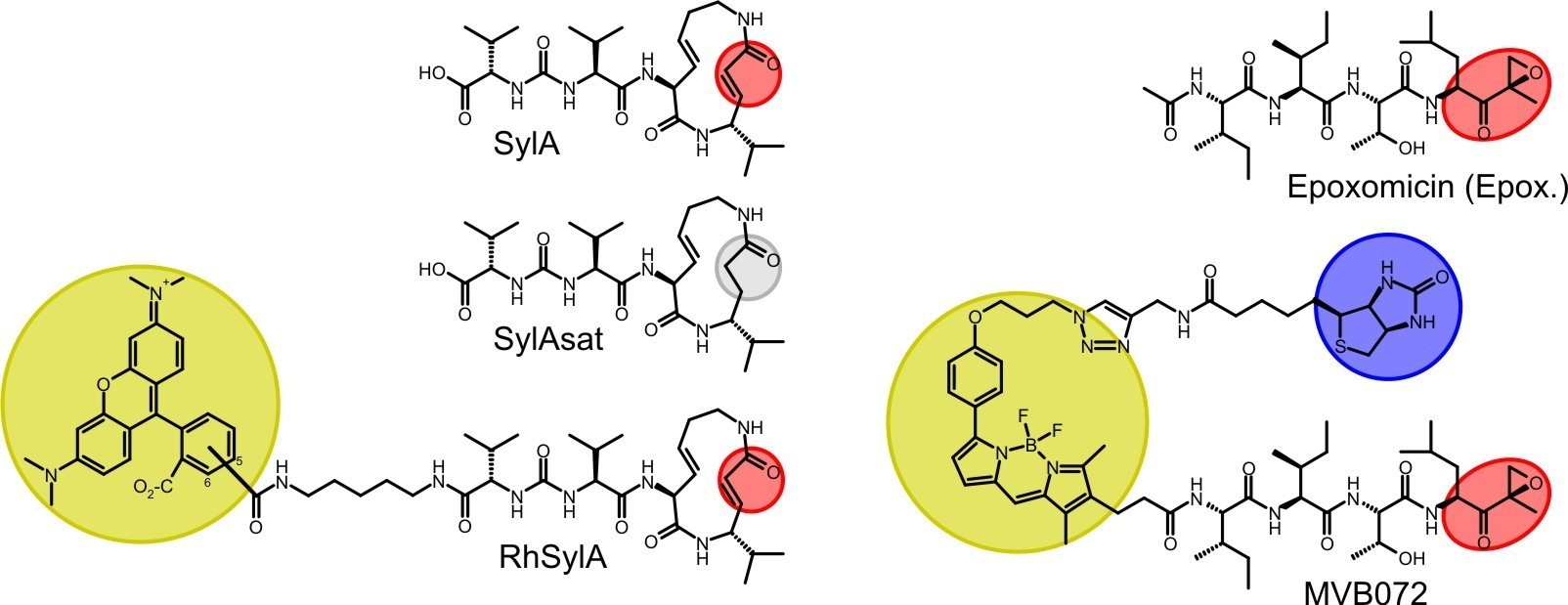

Supplement: Figure S2 — Structures of chemicals used in this study. Reactive groups (red), biotin (blue) and fluorescent reporter (yellow). (JPG) [file ppat.1003281.s002.jpg]

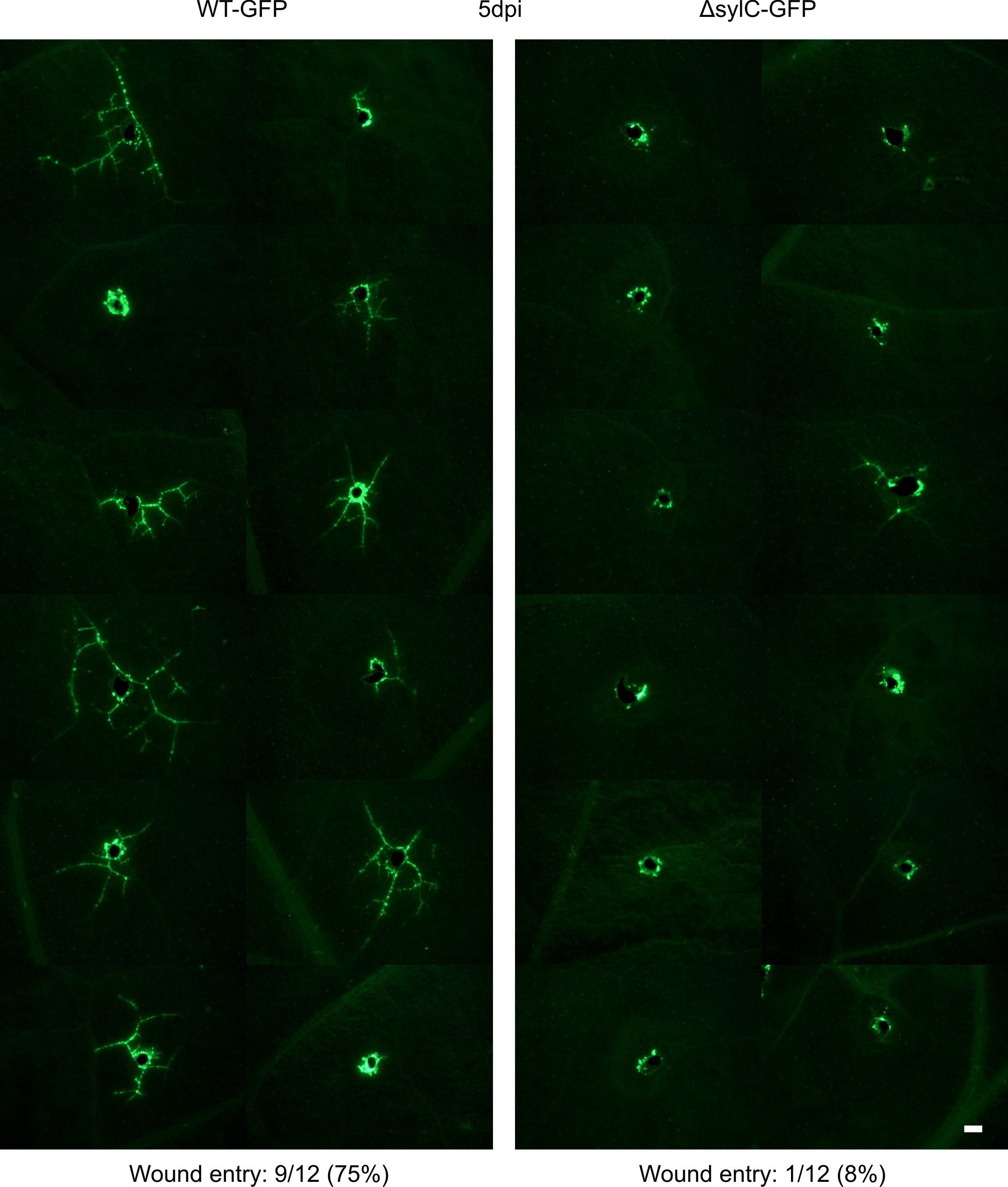

Supplement: Figure S3 — Representative wound entry assay experiment. WT-GFP and ΔsylC-GFP bacteria were toothpick-inoculated from a fresh plate into different leaves of different plants of N. benthamiana. Pictures were made at 5 dpi using stereo fluorescence microscopy using identical settings. The frequency of host entry at each wound inoculation sites was counted over 12 toothpick sites, as shown at the bottom. Scale bar, 1 mm. (JPG) [file ppat.1003281.s003.jpg]

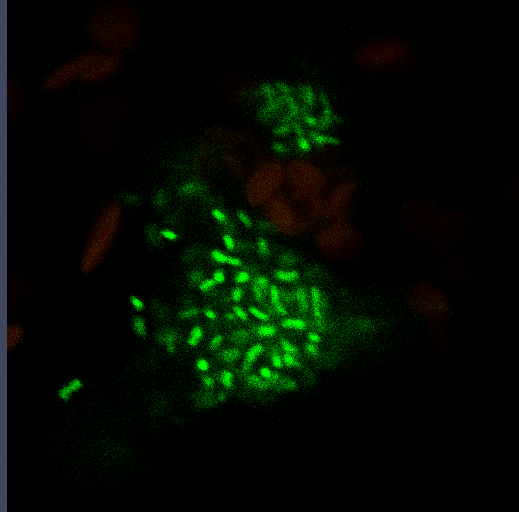

Supplement: Movie S9 — Bacterial motility at 24 hpi in sylC-GFP colonies with 50 µM SylA. Leaves were infiltrated with 105 ΔsylC-GFP bacteria/mL containing 50 µM SylA and bacterial colonies were imaged at 24 hpi by confocal microscopy. The length of the movie is 100 seconds. The size of the movie frame corresponds to 512×512 µm. (GIF) [file ppat.1003281.s012.gif]

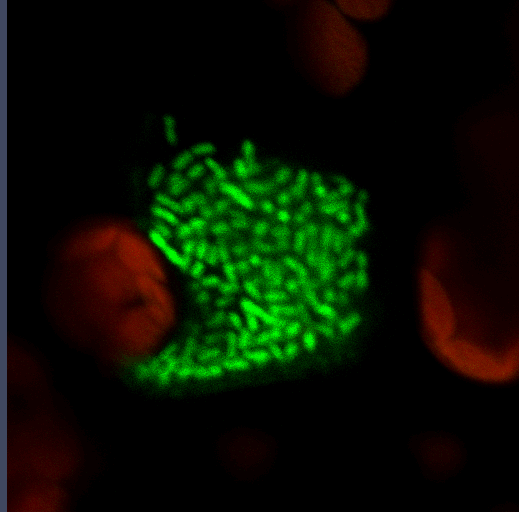

Supplement: Movie S10 — Bacterial motility at 24 hpi in sylC-GFP colonies with 0.25% DMSO. Leaves were infiltrated with 105 ΔsylC-GFP bacteria/mL containing 0.25% DMSO and bacterial colonies were imaged at 24 hpi by confocal microscopy. The length of the movie is 100 seconds. The size of the movie frame corresponds to 512×512 µm. (GIF) [file ppat.1003281.s013.gif]
