# Supplementary material for: Pseudomonas syringae pv. syringae Uses Proteasome Inhibitor Syringolin A to Colonize from Wound Infection Sites
Source: PLoS Pathog. 2013 Mar 28;9(3):e1003281. doi: 10.1371/journal.ppat.1003281 (PMC3610659; doi:10.1371/journal.ppat.1003281)
Supplement: Table S1 — Identification of MVB072-labeled proteins from N. benthamiana . (PDF) [file ppat.1003281.s014.pdf]

Table S1 Identification of MVB072-labeled Proteins from *N. benthamiana*

peptide	XCorr	DeltCN	ZScore	Ion%	spectra	charge
**β1: Nbentha|TC11080 12 peptides, 14 spectral counts, 35.6% sequence coverage**						
R.TSTGMYVANR.A	3.1733	0.3181	5.095	94.4%	2	2+
R.ASDKITQLTDNVYVCR.S	3.5302	0.1872	4.68	41.7%	1	3+
R.YFLHQHTIQLGQPATVK.V	4.7218	0.3395	7.215	59.4%	1	2+
R.YFLHQHTIQLGQPATVK.V	4.9673	0.3392	6.247	50.0%	1	3+
K.AMLQTGMIIGGWDK.Y	4.4574	0.2942	6.828	80.8%	1	2+
K.AM*LQTGMIIGGWDK.Y	2.9044	0.126	3.636	69.2%	1	2+
K.AM*LQTGM*IIGGWDK.Y	4.1316	0.2955	5.897	80.8%	1	2+
M.LQTGMIIGGWDK.Y	3.4043	0.3253	5.905	86.4%	1	2+
M.LQTGM*IIGGWDK.Y	4.0077	0.3047	6.249	86.4%	1	2+
M.LQTGM*IIGGWDKYEGGK.I	3.852	0.181	5.498	53.1%	1	2+
R.EGM*TQEEAEKLVVTAVSLAIAR.D	3.7147	0.0842	4.464	32.1%	1	3+
K.LVVTAVSLAIAR.D	4.4324	0.2351	4.766	90.9%	2	2+
**β2: tobacco|TC14992 3 peptides, 3 spectral counts, 19.4% sequence coverage**						
I.HYMAPNIYCCGAGTAADTEAVTDMVSSQLK.L	5.2152	0.3178	6.197	25.9%	1	3+
R.VVTALTLLK.S	2.756	0.0977	4.535	87.5%	1	2+
R.EIVQVIEGGDAM*EE.-	4.3334	0.3787	6.75	84.6%	1	2+
**β5: tobacco|TC16354 23 peptides, 27 spectral counts, 52.4% sequence coverage**						
K.GGVMVAADSR.A	2.169	0.1954	5.039	61.1%	1	1+
K.GGVMVAADSR.A	3.2103	0.2612	5.324	77.8%	1	2+
R.ASMGGYISSQSVK.K	4.2853	0.2594	5.313	79.2%	2	2+
R.ASM*GGYISSQSVK.K	3.9103	0.3064	6.33	83.3%	2	2+
R.GMGLSVGTMIAGWDEK.G	4.8122	0.3413	7.835	76.7%	1	2+
R.GMGLSVGTM*IAGWDEK.G	4.1475	0.3178	6.532	66.7%	1	2+
R.GM*GLSVGTMIAGWDEK.G	4.7004	0.2269	6.312	76.7%	1	2+
R.GM*GLSVGTM*IAGWDEK.G	4.2872	0.2864	5.697	73.3%	1	2+
R.GMGLSVGTMIAGWDEKGPGLYYVDSEGGR.L	3.7003	0.2982	5.315	24.1%	1	3+
R.GMGLSVGTM*IAGWDEKGPGLYYVDSEGGR.L	5.7723	0.4083	7.129	33.9%	1	3+
R.GM*GLSVGTMIAGWDEKGPGLYYVDSEGGR.L	4.2721	0.2698	5.964	26.8%	1	3+
R.GM*GLSVGTM*IAGWDEKGPGLYYVDSEGGR.L	5.7573	0.3491	7.041	35.7%	1	3+
K.GPGLYYVDSEGGR.L	3.8079	0.3303	6.698	79.2%	1	2+
R.FSVGSGSPYAYGVLDSGYR.F	3.4172	0.1948	4.889	38.9%	1	3+
R.FSVGSGSPYAYGVLDSGYR.F	5.8757	0.4713	8.962	63.9%	2	2+
R.FDLSVEEAAELAR.R	4.4415	0.3781	7.56	79.2%	1	2+
R.AIYHATFR.D	1.997	0.0496	3.574	64.3%	2	2+
R.DGASGGVASVYHVGPNGWK.K	3.232	0.1516	4.445	55.6%	1	2+
R.DGASGGVASVYHVGPNGWK.K	3.249	0.1813	5.064	41.7%	1	3+
K.KLSGDDVGELHYNYYPVELESVEQEMAEVPVA.-	3.6705	0.2459	5.256	25.0%	1	3+
K.KLSGDDVGELHYNYYPVELESVEQEM*AEVPVA.-	3.0121	0.3297	5.612	22.6%	1	3+
K.LSGDDVGELHYNYYPVELESVEQEMAEVPVA.-	3.9754	0.3128	5.795	23.3%	1	3+
K.LSGDDVGELHYNYYPVELESVEQEM*AEVPVA.-	4.0321	0.3729	6.439	27.5%	1	3+
