# Supplementary material for: Pseudomonas syringae pv. syringae Uses Proteasome Inhibitor Syringolin A to Colonize from Wound Infection Sites
Source: PLoS Pathog. 2013 Mar 28;9(3):e1003281. doi: 10.1371/journal.ppat.1003281 (PMC3610659; doi:10.1371/journal.ppat.1003281)
Supplement: Table S2 — Plasmids and strains used in this study. (PDF) [file ppat.1003281.s015.pdf]

Table S2 Plasmids and strains used in this study.

**Plasmid**	**Description**	**Resistance***	**Reference**
pFK78	pML123 expressing GFP	Gm	This work
pML123	Broad host range cloning vector	Gm	Labes et al., 1990
pPL3syl (p63::syl)	Cosmid carrying *sylA-E* of *Psy*B301D-R	Tet	Ramel et al. 2009
**Strain**	**Description**	**Resistance***	**Reference**
E.coli	Triparental mating pRK600	Cm	R. Dudler
*Psy*B728a-WT	Isolated from snap beans	Rif	Loper & Lindow, 1999
*Psy*B728a-ΔsylC	ΔsylC mutant of *Psy*B728a-WT	RifTet	Groll et al., 2008
*Psy*B728a-WT-GFP	*Psy*B728a-WT carrying pFK78	RifGm	This work
*Psy*B728a-ΔsylC-GFP	*Psy*B728a-ΔsylC carrying pFK78	RifTetGm	This work
*Psy*B301D	Isolated from snap beans	Rif	Amrein et al., 2004
*Psy*B301D ΔsylC	ΔsylC mutant of *Psy*B301D-R	RifTet	Amrein et al., 2004
*Psy*B301D ΔsylD	ΔsylD mutant of *Psy*B301D-R	RifTet	Amrein et al., 2004
*Psy*B301D ΔsylA-E	ΔsylA-E cluster deletion mutant of *Psy*B728a	Rif	Ramel et al., 2009
*Psy*B301D ΔsylA-E(pPL3syl)	*Psy*B301D ΔsylA-E carrying pPL3syl	RifTet	Ramel et al., 2009
*Psy*B301D-GFP	*Psy*B301D carrying pFK78	RifGm	This work
*Psy*B301D ΔsylC-GFP	*Psy*B301D ΔsylC carrying pFK78	RifTetGm	This work
*Psy*B301D ΔsylD-GFP	*Psy*B301D ΔsylD carrying pFK78	RifTetGm	This work
*Psy*B301D ΔsylA-E-GFP	*Psy*B301D ΔsylA-E carrying pFK78	RifGm	This work
*Psy*B301D *Δsyl(pPL3syl)*-GFP	*Psy*B301D ΔsylA-E carrying pPL3syl and pFK78	RifTetGm	This work

* Cm, Chloramphenicol; Gm, Gentamycin; Rif, Rifampicin; Tet, Tetracyclin

Amrein, H., Makart, S., Granado, J., Shakya, R., Schneider-Pokorny, J., and Dudler, R. (2004) Functional analysis of genes involved in the synthesis of syringolin A by *Pseudomonas syringae* pv. *syringae* B301D-R. *Mol. Plant-Microbe Interact.* **17**, 90-97.

Groll, M., Schellenberg, B., Bachmann, A. S., Archer, C. R., Huber, R., Powell, T. K., Lindow, S., Kaiser, M., and Dudler, R. (2008) A plant pathogen virulence factor inhibits the eukaryotic proteasome by a novel mechanism. *Nature* **452**, 755-758.

Hwang, M. S. H., Morgan, R. L., Sarkar, S. F., Wang, P. W., and Guttman, D. S. (2005). Phylogenetic characterization of virulence and resistance phenotypes of *Pseudomonas syringae*. *Appl. Environ. Microbiol*. **71**, 5182-5191.

Labes M., Pühler A., and Simon R. (1990) A new family of RSF1010-derived expression and lac-fusion broad-host-range vectors for gram-negative bacteria. *Gene* **89**, 37-46.

Loper, J. E., and Lindow, S. E. (1987) Lack of evidence for *in situ* fluorescent pigment production *by Pseudomonas syringae* pv. *syringae* on bean leaf surfaces. *Phytopathol*. **77**, 1449-1454.

Ramel, C., Tobler, M., Meyer, M., Bigler, L., Ebert, M. O., Schellenberg, B., and Dudler, R. (2009) Biosynthesis of the proteasome inhibitor syringolin A: the ureido group joining two amino acids originates from bicarbonate. *BMC Biochemistry* **10***:*26
